# On‐Surface Synthesis and Characterization of Tetraazanonacene

**DOI:** 10.1002/anie.202504707

**Published:** 2025-07-09

**Authors:** Zilin Ruan, Liping Ye, Yogendra Singh, Tim Naumann, Faming Kang, Ye Liu, Michael Mastalerz, J. Michael Gottfried

**Affiliations:** ^1^ Department of Chemistry Philipps University Marburg 35037 Marburg Germany; ^2^ Organisch‐Chemisches Institut Ruprecht‐Karls‐Universität Heidelberg 69120 Heidelberg Germany

**Keywords:** Azaacene, Noncontact atomic force microscopy, On‐surface synthesis, Open‐shell structures, Scanning tunnelling microscopy

## Abstract

Isosteric replacement of CH units of acenes by nitrogen induces significant changes in their electronic, redox, and spectroscopic properties. Here, we describe the on‐surface synthesis of a nonacene analogue substituted with four nitrogen atoms on a Au(111) surface by a two‐step atom manipulation, employing a bis vinylene‐bridged precursor synthesized in solution. The generated tetraazanonacene has been investigated by scanning tunnelling microscopy/spectroscopy (STM/STS) and noncontact atomic force microscopy (nc‐AFM), combined with first‐principles calculations. We found that, compared to the pristine nonacene, the electronegative nitrogen atoms lower the frontier orbitals, resulting in an increased STS transport gap of 1.49 eV. Furthermore, the formation of four pyridine‐like rings induces a stronger modulation of the aromaticity in tetraazanonacene compared to substitution patterns where nitrogen atoms form pyrazine‐like rings. This observation is different from the nucleus independent chemical shift investigation of previous reported tetraazaundecacene, for which a stronger open‐shell character is expected. Our work provides access to the synthesis of extended azaacenes and to an understanding of their properties.

## Graphical abstract

Tetraazanonacene featuring four isolated pyridine‐like rings was synthesized on Au(111) via STM tip manipulation. Its properties were characterized using scanning tunneling microscopy and spectroscopy, density functional theory, and multireference calculations. The study highlights how the distribution of nitrogen atoms influences the electronic structure and aromaticity of azaacenes.

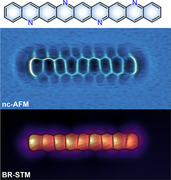

## Introduction

Acenes are a fascinating class of polycyclic aromatic hydrocarbons (PAH) that have gained considerable research interest due to their potential application in organic electronics as well as their role in advancing fundamental knowledge about conjugated aromatic systems.^[^ ^1^, ^2^ ^]^ Isosteric exchange of CH units of the acenes by nitrogen atoms formally give azaacenes. The nitrogen substitution pattern, i.e., the number, position, and type of nitrogen atoms, plays an important role in precise tuning of frontier orbital energy level alignment and radical characters, as has been theoretically investigated.^[^ ^3^, ^4^, ^5^ ^]^ The resulting improved resistance to oxidation, photodegradation, and cyclodimerization^[^ ^6^ ^]^ make azaacenes potential n‐type organic semiconducting materials. Indeed, smaller azaacenes (such as substituted tetraazapentacenes) have been studied in this respect over the past three decades, achieving high electron mobilities in thin film transistors.^[^ ^7^, ^8^, ^9^, ^10^, ^11^, ^12^ ^]^ Like their parent acenes, longer azaacenes exhibit poor chemical stability, which is attributed to a substantial degree of diradical or polyradical character. As a result, synthesizing unsubstituted azaacenes longer than pentacene remains a significant challenge.^[^ ^13^, ^14^, ^15^, ^16^, ^17^ ^]^ Substituted azahexacene,^[^ ^18^, ^19^ ^]^ azaheptacene,^[^ ^20^, ^21^ ^]^ and even azaoctacene in its reduced form^[^ ^22^ ^]^ have been synthesized in solution by introducing bulky groups (e.g., silylethynyl groups) at the molecular backbone. To the best of our knowledge, azaacenes with more than seven rings, particularly in their unsubstituted form, have only been reported to be generated on surfaces.^[^ ^23^ ^]^

In recent years, on‐surface synthesis has enabled the generation of larger unsubstituted acenes, providing pathways to explore their rich physical and chemical properties at the molecular scale.^[^ ^24^, ^25^, ^26^, ^27^, ^28^, ^29^, ^30^ ^]^ This approach holds also promise for studying longer azaacenes. In particular, tetraazaundecacene, which contains two pyrazine rings, and its reduced form have been synthesized on a Au(111) surface.^[^ ^23^ ^]^ The synthesized tetraazaundecacene shows considerable open‐shell character compared to the pristine undecacene. In addition, its reduced form has a closed‐shell electronic ground state and exhibits local anti‐aromaticity. Compared to acenes, azaacenes offer significantly greater structural diversity—and, consequently, more tunable electronic properties—due to the possible variations in the number and positions of embedded nitrogen atoms; however, despite this versatility, the electronic properties of larger azaacenes with different lengths and nitrogen substitution patterns have so far only been explored theoretically.^[^ ^3^, ^4^, ^5^ ^]^

Here, we report the synthesis of tetraazanonacene using a combined in‐solution and on‐surface approach, and the detailed investigation of structural and electronic properties by scanning tunneling microscopy/spectroscopy (STM/S), and noncontact atomic force microscopy (nc‐AFM), along with density functional theory (DFT) and multireference calculations. The on‐surface generation was achieved by forced aromatization of a rationally designed stable chiral vinylene bridged nonacene precursor obtained by chirality‐assisted synthesis (CAS),^[^ ^31^ ^]^ through tip manipulation in a low‐temperature STM. The experimental and theoretical characterizations of tetraazanonacene were studied to get an insight into its electronic properties and aromaticity, which is presented herein.

## Results and Discussion

Previously, the Friedländer condensation has been used for chirality‐assisted synthesis to construct nanobelt in very high yields.^[^ ^32^ ^]^ As the successful formation of two pyridine rings in different directions between enantiopure bicyclodione and dibenzoyl‐diamine, we envisioned that chiral and enantiopure bicyclodione **1** ^[^ ^33^, ^34^ ^]^ and diimine‐dialdehyde **2** will allow the synthesis of azaacene precursors with alternative arrangement of pyridine rings, which differ from those azaacenes consisting of pyrazine rings formed either by imine condensation^[^ ^21^, ^23^ ^]^ or by Buchwald–Hartwig amination followed by oxidation reaction.^[^ ^18^, ^19^, ^20^ ^]^ Moreover, CAS strategy is an efficient tool to construct one‐handed propagation of curvature, thus giving exclusively the configuration with both bridges on the same side in high yield. Chiral bicyclodione (*R*,*R*)‐**1** ^[^ ^33^, ^34^ ^]^ was reacted with compound **2**,^[^ ^35^ ^]^ which acts as a surrogate of unstable 2,5‐diaminoterephthalaldehyde, with diphenyl phosphate in toluene gave (*R,S,R,S*)‐**3** in 27% yield. To mitigate the loss of starting material **2**, a stirred solution of **2** in toluene was slowly added to the toluene solution containing (*R,R*)‐**1**. Subsequently, a second Friedländer condensation of (*R,S,R,S*)‐**3** with 2‐aminobenzaldehyde formed precursor (*R,S,R,S*)‐**4** in 91% yield.

**Figure 1:**
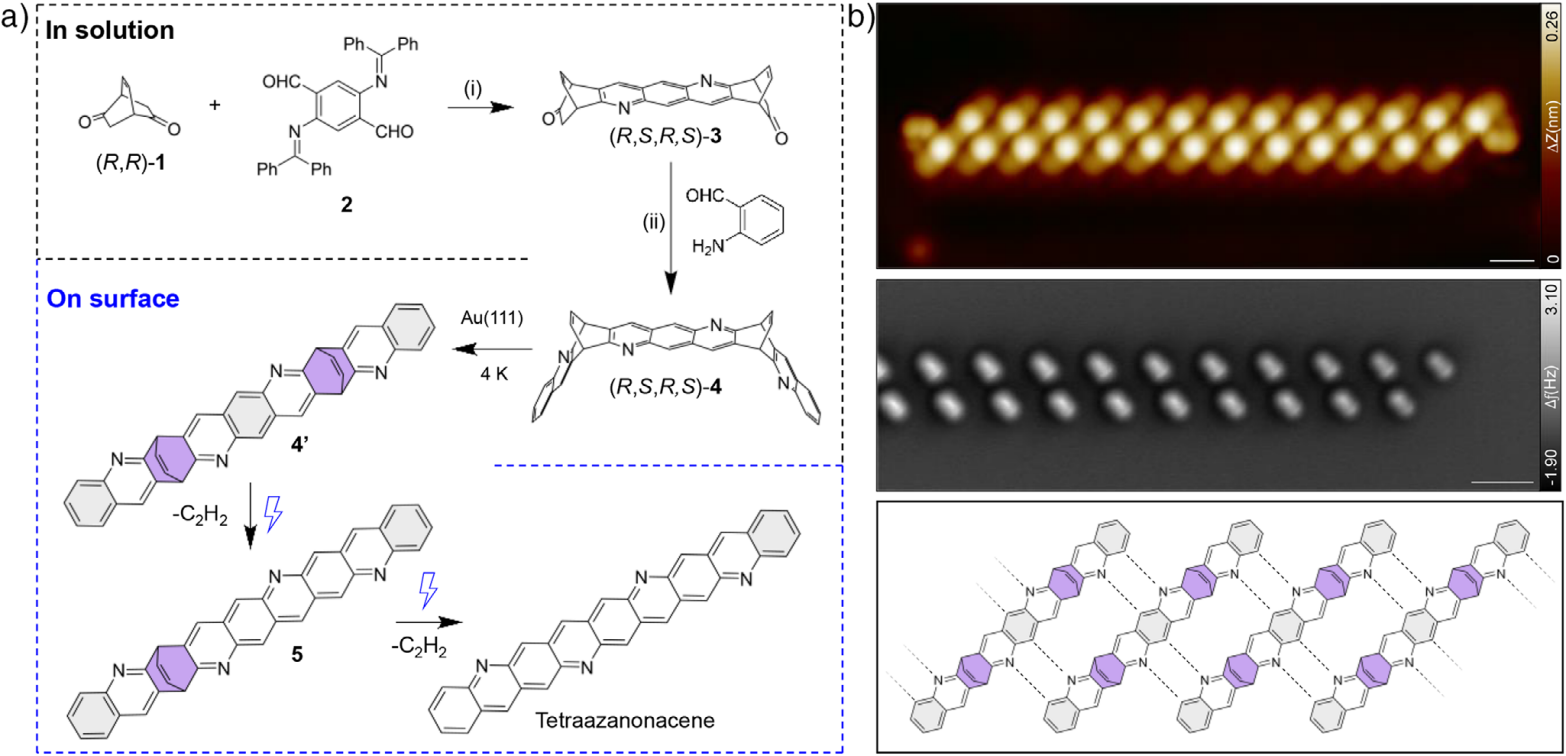
Generation of tetraazanonacene on Au(111). a) Scheme of the combined in‐solution and on‐surface synthesis of tetraazanonacene. The enantiopure precursor **4** is synthesized in solution, reagents, and conditions: i) diphenyl phosphate, toluene, reflux, overnight, 27%; ii) diphenyl phosphate, mesitylene, 165 °C, overnight, 91%. **4** was then sublimed onto the Au(111) surface under UHV for tip manipulation at 4 K. b) (top) STM and (middle) nc‐AFM image of the chain‐like molecular self‐assembly of **4** on Au(111), which is stabilized by intermolecular ─N⋯H‐C interactions (bottom). Scanning parameters: (b) STM, *V*_s_ = 0.15 V, *I*_t_ = 20 pA; nc‐AFM, *V*s = 10 mV. Scale bars: 1 nm.

**Figure 2:**
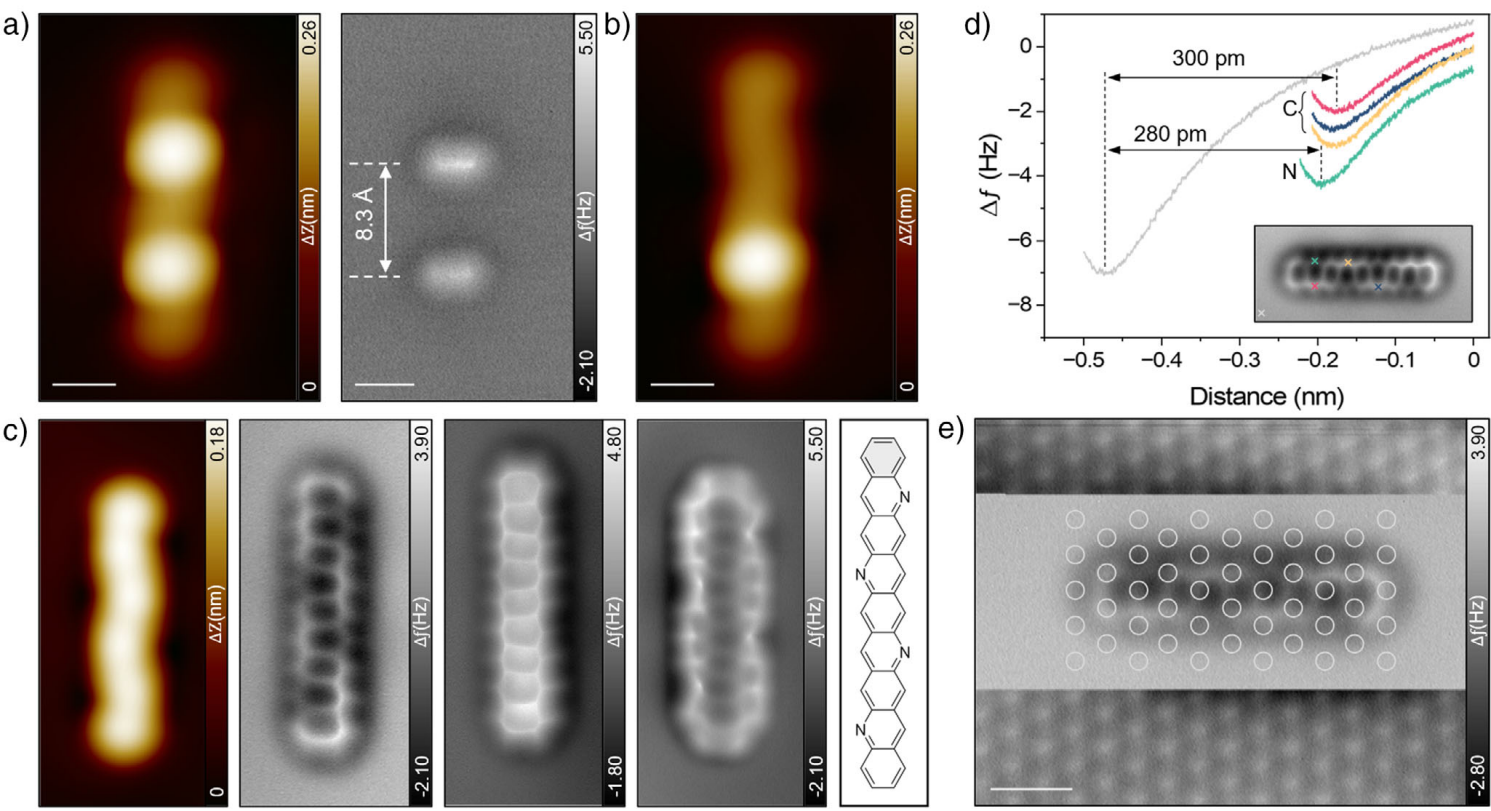
Structural characterization of tetraazanonacene. a) (left) STM and (right) nc‐AFM image of an isolated precursor **4**. b) STM image of the intermediate after removing one etheno bridge. c) Characterization of tetraazanonacene (left to right): STM image, two constant‐height nc‐AFM images acquired at different heights, constant current nc‐AFM image, and corresponding chemical model of tetraazanonacene. The images were obtained directly after removal of the second etheno bridge. d) Frequency versus tip‐height curves measured at the spots marked in the inset. e) Variable‐height nc‐AFM image with the atomic resolution of both the molecule and the Au(111) surface. Scanning parameters: (a) and (b) STM, *V*s = 0.15 V, *I*_t_ = 20 pA; nc‐AFM, *V*s = 10 mV. c)–e) STM, *V*s = 0.15 V, *I*_t_ = 30 pA; nc‐AFM, *V*s = 10 mV. Scale bars: (a) and (b) 1 nm; (c) and (d) 1.5 × 3.5 nm^2^; (e) 0.5 nm.

Enantiopure **4** was then vapor deposited from a standard Knudsen cell heated to 520 K, onto a Au(111) surface held at room temperature in ultra‐high vacuum (UHV) conditions. The sample was then transferred to the STM operated at 4 K for characterization and manipulation. The STM image shown in Figure 1b depicts molecular self‐assembly of intact **4** (Figure S32). Upon adsorption, enantiopure **4** adopts a preferred geometry in which the bridges point away from the surface. The presence of two vinylene groups that give rise to the two bright protrusions in STM image is confirmed by the bright feature in the CO‐tip nc‐AFM image (Figure 1b, middle) resulting from the CO tilting^[^ ^36^ ^]^ due to its interaction with the hydrogen substituents at the vinylene bridges. Overlaying the molecular structure with the high‐resolution images indicates that the self‐assembly is stabilized by ─N⋯H‐C interactions between adjacent molecules (Figure 1b, bottom). Figure 2a shows the high‐resolution STM image of a single precursor molecule, isolated by lateral tip manipulation, in which the nitrogen‐induced distortion is partially visible at the edge. The corresponding nc‐AFM image reveals a distance of 8.3 Å between two vinylene bridges (see also Figure S34), which is shorter than that for the structurally similar precursor for pentadecacene (9.3 Å).^[^ ^30^ ^]^ The shorter distance may be explained by a contraction of the conjugated C─N bond compared to a conjugated C─C bond (in a similar molecular environment), as well as by the altered molecule‐surface interactions resulting from the incorporated nitrogen atoms. Following the established strategy for synthesizing long acenes on surfaces,^[^ ^29^, ^30^ ^]^ we conducted tip manipulation to eliminate the vinylene bridges, thereby inducing aromatization. Figure 2b, shows the STM image of the monovinylene bridged intermediate after removing one protecting group, which reduces the number of Clar's sextets from three to two. A second manipulation step eliminates the remaining vinylene bridge, generating a planar species with a noticeable indentation feature at the edges in the STM image (Figure 2c), presumably corresponding to tetraazanonacene. We also attempted to remove the etheno‐protecting groups by annealing; however, this treatment caused substantial fragmentation and subsequent desorption (Figure S33), as previously observed for a chemically similar precursor. ^[^ ^23^ ^]^

To characterize the chemical structure of the obtained species, we performed nc‐AFM measurement using a CO‐functionalized tip. The variable‐height nc‐AFM images shown in Figure 2c reveal the annulated rings, confirming the successful generation of tetraazanonacene on the Au(111) surface (see also Figure S34). Notably, every second ring, where the nitrogen is incorporated, appears slightly larger. This observation can be explained by strain induced by different bond lengths and altered molecule‐surface interaction due to the nitrogen doping.

The corresponding nc‐AFM image acquired in constant current mode (Figure 2c, right) clearly shows more pronounced features at the nitrogen sites, as well as the absence of any hydrogen atoms at the nitrogen, confirming the successful generation of tetraazanonacene. The frequency shift versus tip‐height curves acquired at different sites (Figure 2d) indicate that tetraazanonacene has an apparent adsorption height of around 300 pm, suggesting weak interaction with the surface, which is in line with the observed very high mobility of tetraazanonacene (Figure S35). The minima for the nitrogen atoms lie at slightly lower apparent heights than the carbon atoms by around 20 pm, and thus, they are less visible in the constant‐height images. The variable‐height nc‐AFM image, which provides atomic resolution of both the surface and the molecule (Figure 2e), shows that tetraazanonacene aligns approximately with the [112 ®] direction of the surface, while the nitrogen atoms are pinned to the hollow sites.

**Figure 3:**
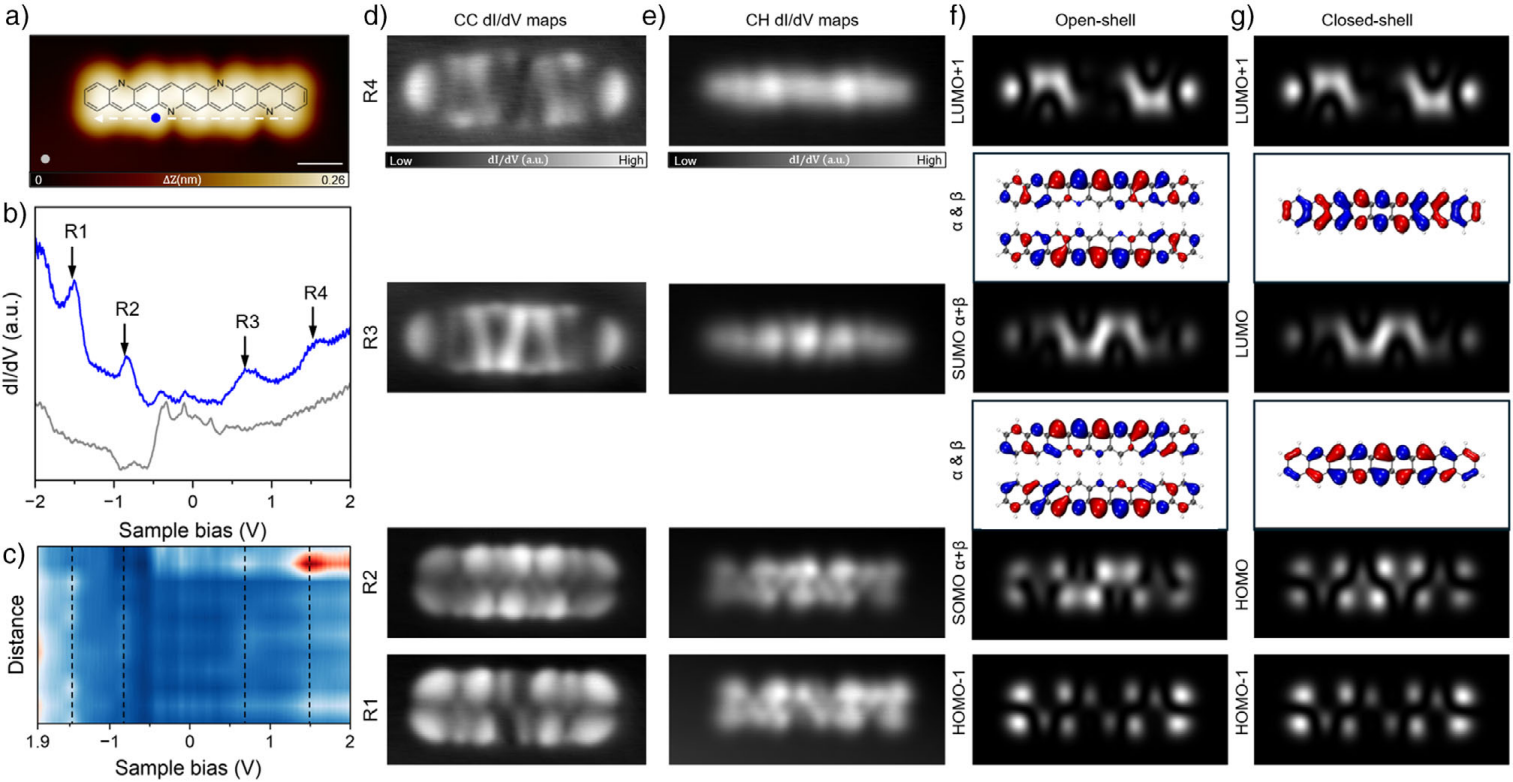
Electronic properties of tetraazanonacene. a) STM image of occupied state of tetraazanonacene. b) dI/dV point spectra acquired at the blue and grey spots marked in (a). Four resonance peaks R1 (−1.45 V), R2 (−0.83 V), R3 (0.66 V), and R4 (1.58 V) are indicated by arrows. c) 2D color map of 10 dI/dV curves acquired along the edge of tetraazanonacene indicated by white arrow in (a), peaks R1‐R4 are marked by black dashed lines. d) Experimental constant current and e) constant height dI/dV maps taken at the four peaks in (b). f) DFT calculated constant height (6 Å above the molecular plane) dI/dV maps and orbitals for open‐shell solution and g) closed‐shell solution. (a) *V*_s_ = −1.45 V, *I*_t _= 50 pA. Scale bars: (a) 1 nm; (d) and (e) 1.5 × 3.5 nm^2^.

Next, we conducted STS measurements to probe the electronic properties of tetraazanonacene. Figure 3a shows the STM image of the occupied states of tetraazanonacene, in which the nitrogen sites are clearly visible. The dI/dV curve measured on tetraazanonacene (Figure 3b) exhibits resonance peaks at approximately −0.83 and 0.66 V (with an error of ± 0.02 eV), corresponding to the highest occupied (HOMO) and the lowest unoccupied molecular orbitals (LUMO), respectively. This results in an experimental STS transport gap of ∼1.49 eV. In addition, two peaks at −1.45 and 1.58 V (with an error of ± 0.02 eV), assigned to HOMO‐1 and LUMO +1, respectively, are also identified (see also Figure S36). The spatial distribution of these states is visualized by dI/dV maps measured at the corresponding energies in constant current (Figure 3d) and constant height mode (Figure 3e). The substituted nitrogen at the opposite edges of every second ring leads to only rotational symmetry of the molecule, which is also reflected in the distributions of the orbitals measured here. The obtained transport gap for tetraazanonacene (1.49 eV) shows a significant increase compared to its nonacene (1.19 eV) analogue.^[^ ^24^ ^]^ This can be explained by the different stabilization effect on the frontier orbitals^[^ ^3^ ^]^ Specifically, the electron‐withdrawing nitrogen has more pronounced influence on the occupied states, causing a more significant stabilization of the HOMO (∆*E *= 0.49 eV) than that of the LUMO (∆*E *= 0.19 eV), compared to nonacene.^[^ ^24^ ^]^

These observations are further supported by DFT calculations (B3LYP/6–311 + G(d,p)) performed for the gas phase molecule, which appears justified considering the very weak interaction between tetraazanonacene and the surface, as discussed previously. Theoretically, both nonacene^[^ ^13^ ^]^ and tetraazanonacene exhibit an open‐shell singlet ground state (Table S1 and Figure S37). However, the energy difference between the closed‐shell and open‐shell configurations is reduced from 0.27 eV in nonacene to 0.18 eV in tetraazanonacene. Simulated dI/dV maps based on the broken‐symmetry open‐shell singlet solution (Figure 3f) and the closed‐shell singlet solution (Figure 3g) reproduce the experimentally observed orbital features. A key qualitative difference lies in the continuous lobes of the SOMO (Figure 3f) versus the distinct nodal gaps between lobes in the HOMO (Figure 3g). However, the latter nodal pattern is less pronounced in the experimental dI/dV maps obtained in both constant‐current and constant‐height modes, suggesting a possible open‐shell contribution to the electronic ground state of tetraazanonacene. To further probe the electronic structure, we have also performed more accurate multireference complete active space self‐consistent field (CASSCF) calculations for both tetraazanonacene and nonacene (Tables S2 and S3 and Figures S38 and S39). These predict a diradical character in both molecules, though it is weaker in tetraazanonacene. This finding is consistent with the experimentally observed stabilization of the frontier orbitals. Moreover, the occasionally observed formation of a gold complex (Figure S40) points to the high reactivity of tetraazanonacene, likely a result of its open‐shell diradical nature, further supporting the presence of an open‐shell ground state. It is worth noting that for longer acenes (>dodecacene), the influence of the surface is important for interpreting their STS transport gap and electronic ground state.^[^ ^28^, ^29^ ^]^ However, for acenes and azaacenes with lengths comparable to that of tetraazanonacene, gas‐phase calculations show good agreement with experimental findings,^[^ ^23^, ^24^ ^]^ further justifying the chosen theoretical model.

**Figure 4:**
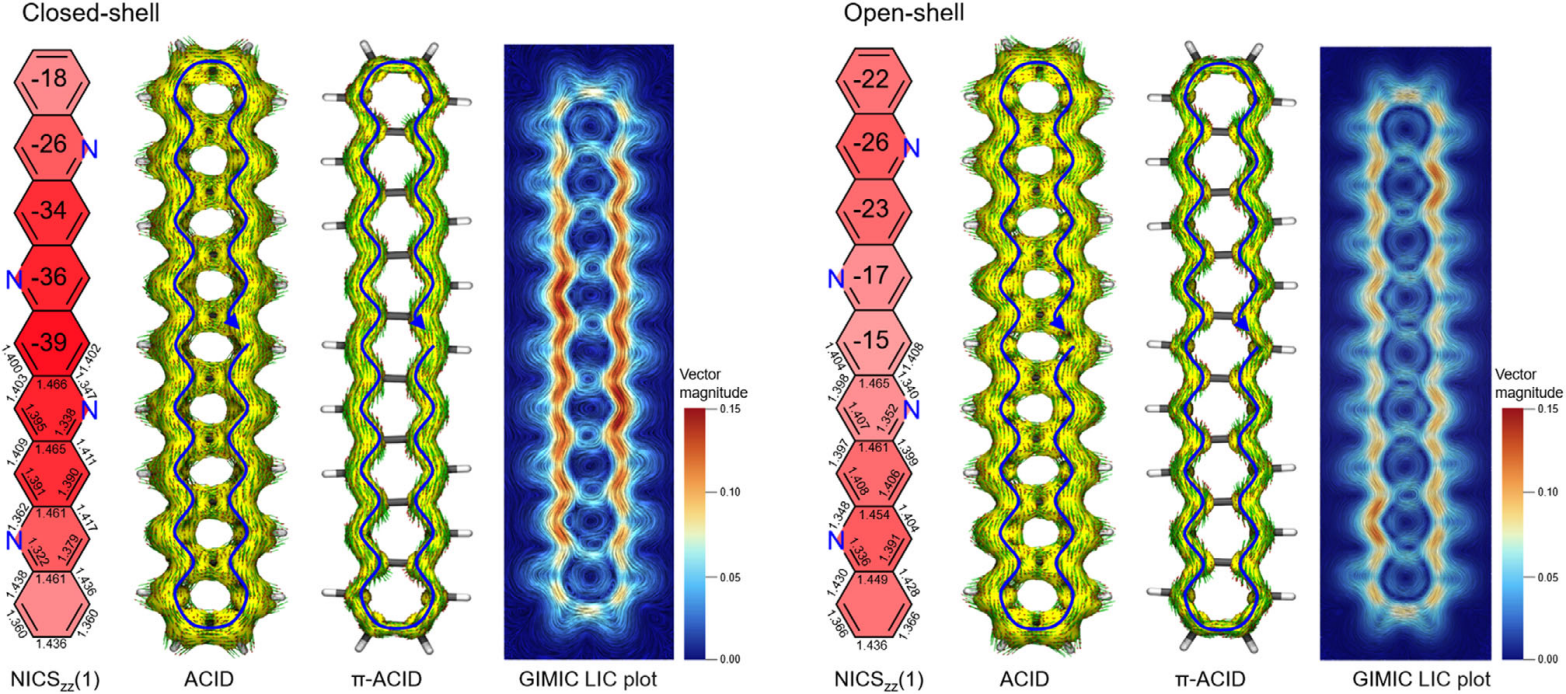
Computational characterization of aromaticity of tetraazanonacene. NICS*_zz_* (1) patterns, ACID and π‐ACID, GIMIC LIC plot of tetraazanonacene for (left) closed‐shell and (right) open‐shell solution. The current density for GIMIC LIC plot is obtained at 1 Bohr above the molecular plane, the color corresponds to the magnitude of induced current.

In order to further characterize the π‐system and the impact of the unique alternating nitrogen substitution, we calculated the NICS*_zz_* (1) variant for both the closed‐shell and open‐shell solutions using the nucleus independent chemical shift (NICS) method.^[^ ^37^ ^]^ The results reveal that, in the closed‐shell solution, the central region of tetraazanonacene exhibits strong aromaticity, which decreases significantly in the open‐shell solution (Figure 4). Conversely, the terminal rings display the opposite trend, showing enhanced aromaticity in the open‐shell case. These observations align with findings for shorter acenes^[^ ^38^, ^39^, ^40^, ^41^ ^]^ and acenes of similar size to tetraazanonacene,^[^ ^42^ ^]^ but contrast with those for a recently reported tetraazaundecacene, where the four nitrogen atoms are contained in two pyrazine units.^[^ ^23^ ^]^ We attribute this inconsistency not to differences in spin contamination arising from the distinct open‐shell character of the two large azaacenes, but possibly an improper handling of the electronic ground state for obtaining NICS*_zz_* (1) value.

To gain further insight, we performed anisotropy of the induced current density (ACID) calculations to analyze the induced current (Figure 4).^[^ ^43^ ^]^ The full orbital ACID and π‐ACID patterns for closed‐shell and open shell solutions indicate diatropic currents along the peripheries, confirming the aromatic nature in both cases. To quantitatively visualize the induced current density, we calculated the magnetically induced current density susceptibility using the gauge‐including magnetically induced current (GIMIC) method.^[^ ^44^ ^]^ The line integral convolution (LIC) plot of the magnetically current density, obtained 1 Bohr above the molecular plane, shows diatropic current (see also Figures S41 and S42), consistent with the NICS and ACID analysis. Notably, the closed‐shell solution displays a stronger current density in the central region, whereas the open‐shell solution exhibits enhanced current strength primarily at the terminal rings, particularly near the nitrogen substitutional sites (see also Figures S41 and S42). The unique pyridine‐like nitrogen substitution pattern in tetraazanonacene results in stronger aromaticity compared to the parent nonacene and its isomers, where the four nitrogen atoms are incorporated into two pyrazine‐like units (Figures S43–S45). Importantly, we again find that the longer tetraazaundecacene exhibits aromatic character, as confirmed by multiple aromaticity indicators (Figure S46). These findings underscore the potential of alternating nitrogen substitution patterns to fine‐tune the aromaticity in extended azaacenes.

## Conclusion

In conclusion, we successfully synthesized tetraazanonacene through the two‐step removal of vinylene bridges from a chiral precursor molecule, synthesized with the aid of CAS. The obtained tetraazanonacene was structurally characterized in detail using nc‐AFM. STS measurements of its electronic properties revealed that the incorporation of the more electronegative nitrogen atoms induces a stabilization in the orbital energies, compared to the pristine nonacene, resulting in an increased transport gap of 1.49 eV. DFT calculations indicate considerable open‐shell character of tetraazanonacene, which is further supported by the good agreement between the simulated SOMO orbital and the experimental dI/dV map. Additionally, an aromaticity analysis reveals that in its open‐shell configuration, tetraazanonacene exhibits stronger aromaticity compared to the parent nonacene and other isomers with nitrogen atoms in pyrazine‐like entities. This work represents an important step toward the precise engineering and tailoring of the unique electronic properties of the azaacene family.

## Supporting Information

The authors have cited additional references within the Supporting Information.^[^ ^45^, ^46^, ^47^, ^48^, ^49^, ^50^, ^51^, ^52^, ^53^, ^54^ ^]^

## Conflict of Interests

The authors declare no conflict of interest.

## Supporting information



Supporting Information

## Data Availability

The data that support the findings of this study are available from the corresponding author upon reasonable request.
