# Supplementary material for: On‐Surface Synthesis and Characterization of Tetraazanonacene
Source: Angew Chem Int Ed Engl. 2025 Jul 9;64(33):e202504707. doi: 10.1002/anie.202504707 (PMC12338392; doi:10.1002/anie.202504707)
Supplement: Supplementary file 1 — Supporting Information [file ANIE-64-e202504707-s001.pdf]

Supporting information

On-Surface Synthesis and Characterization of Tetraazanonacene

Zilin Ruan[1], Liping Ye[2], Yogendra Singh[2], Tim Naumann[1], Faming Kang[1], Ye Liu[1], Michael Mastalerz[2], J. Michael Gottfried[1]

[1]*Department of Chemistry, Philipps University Marburg, 35037 Marburg, Germany, michael.gottfried@chemie.uni-marburg.de*
[2]*Organisch-Chemisches Institut, Ruprecht-Karls-Universität Heidelberg, 69120 Heidelberg, Germany, michael.mastalerz@oci.uni-heidelberg.de*

Table of Contents

1. Synthesis 1
2. NMR Spectra 6
3. HR-MS spectra 15
4. IR spectra 16
5. UV-vis and fluorescence spectra 18
6. HPLC Chromatograms 19
7. CD spectra 20
8. Experimental and Theoretical Details and Additional Data 22

1. Synthesis

General: Commercially available reagents were obtained from Chempur, Fisher Scientific, abcr, Alfa Aesar, Sigma-Aldrich or VWR and have been used without further purification unless otherwise mentioned. Thin layer chromatography was performed with POLYGRAM® SIL G/UV254 gel plates by Macherey-Nagel. Detection was accomplished using UV-light (254

nm). Flash column chromatography was performed on silica gel from Macherey-Nagel (particle size: 0.040-0.063 mm) using petroleum ether, dichloromethane, ethyl acetate and their mixtures as eluents. Melting points (not corrected) were measured with a Büchi B-540 melting point analyzer. NMR spectra were taken on a Bruker DRX 300 (300 MHz), Bruker Avance 300 III (300 MHz), Bruker Avance III 400 (400 MHz), Bruker Avance III 500 (500 MHz) and Bruker Avance III 700 (700 MHz) spectrometer. Abbreviations: *s* = singlet, *d* = doublet, *t* = triplet, *m* = multiplet, *br* = broad. Chemical shift values (δ) are expressed in parts per million using residual solvent protons as an internal standard (^{1}H NMR, δ_H = 5.32 for CD_2Cl_2, δ_H = 7.26 for $CDCl_3$; ^{13}C NMR, δ_C = 53.84 for CD_2Cl_2, δ_C = 77.16 for $CDCl_3$). Signals were assigned by 2D NMR experiments (^{1}H-^{1}H COSY, ^{1}H-^{1}H ROESY, ^{1}H-^{13}C HSQC, ^{1}H-^{13}C HMBC). IR-Spectra were recorded on a Bruker Tensor 27 spectrometer on a ZnSe ATR crystal. MALDI-ICR MS experiments were carried out, on a Bruker ApexQe FT-ICR instrument equipped with a 9.4 T superconducting magnet and interfaced to an Apollo II MTP ion source and MALDI-TOF experiments were carried out at a Bruker Autoflex speed MALDI-TOF spectrometer, both with DCTB (trans-2-[3-(4-tert-butylphenyl)-2-methyl-2-propenylidene]malononitrile) as matrix. APCI MS was carried out on a Bruker timsTOFfleX ion mobility-quadrupole-time-of-flight (IM-Q-TOF) spectrometer operated in APCI mode. EI MS measurements have been carried out on a JEOL AccuTOX GCx spectrometer. Elemental analysis was performed by the Microanalytical Laboratory of the University of Heidelberg using an Elementar Vario EL machine. Crystal structure analysis was accomplished on a STOE Stadivari diffractometer with a copper source (λ CuKα = 1.54178 Å) and a PILATUS detector. Data processing and absorption correction (X-Area LANA 1.83.8.0) were accomplished by standard methods. Absorption spectra were recorded on a Jasco UV-VIS V-730. Emission spectra were recorded on a Jasco FP-8300. Specific rotation values were calculated using the Biot equation $[\alpha]_T^\lambda = \frac{\alpha \cdot 100}{l \cdot c}$ with λ = wavelength in nm, T = temperature in °C, α = measured rotation in degrees, l = cuvette path length (1 dm), c = concentration in grams per 100 mL.

2,5-bis((diphenylmethylene)amino)terephthalaldehyde (2)

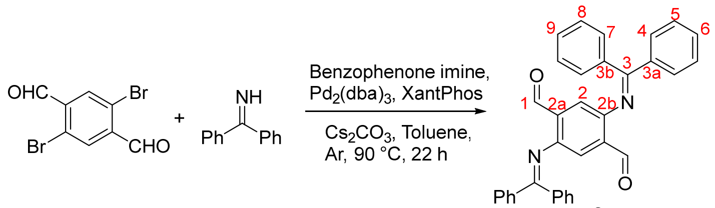

Compound **2** was synthesized according to the literature with some modifications. [1] A mixture of 2,5-dibromo-1,4-benzenedicarboxaldehyde (5.0 g, 17.2 mmol), benzophenone imine (7.2 mL, 42.9 mmol), cesium carbonate (22.3 g, 68.7 mmol), $Pd_2(dba)_3$ (785 mg, 0.86 mmol), and Xantphos (1.49 mg, 2.2 mmol) in toluene (300 mL) was heated in a Schlenk flask under an atmosphere of argon at 90°C for 22 h. The resulting yellow suspension was cooled down to room temperature, H_2O (100 mL) was added, the mixture stirred for 30 min and then the precipitate collected by filtration. The residue was washed with EtOH (3 × 30 mL) and CH_2Cl_2 (30 mL), and dried under a reduced pressure to give **2** (7.644 g, 90 %) as yellow solid (R_f = 0.36 with dichloromethane). **M.p.** = 296-297 °C. **^{1}H NMR** (600 MHz, CD_2Cl_2) δ = 10.2 (s, 2H, H-1), 7.76 (d, J = 7.2 Hz, 4H, H-7), 7.52 (t, J = 7.2 Hz, 2H, H-9), 7.43 (t, J = 7.8 Hz, 4H, H-8), 7.33 (t, J = 7.8 Hz, 2H, H-6), 7.27 (t, J = 7.8 Hz, 4H, H-5), 7.03 (d, J = 7.2 Hz, 4H, H-4), 6.91 (s, 2H, H-2). **^{13}C NMR** (151 MHz, CD_2Cl_2): δ = 190.7 (C-1), 171.2 (C-3), 149.5 (C-2b), 138.9(C-3b), 135.9(C-3a), 131.8(C-9), 130.7 (C-2a), 129.9(C-7), 129.4(C-6), 129.3(C-4), 128.7(C-8), 128.6(C-5), 120.9(C-2). **FT-IR** (neat, ATR): $\tilde{\nu}$ = 2363 cm^{-1} (vs), 3362 (vs), 3312 (vs), 1742 (vs), 1047 (vs), 824 (vs), 1869 (vs), 2023 (vs), 1350 (vs), 1506 (vs), 440 (vs), 3130 (vs), 2773 (vs), 1936 (vs), 1188 (vs), 594 (vs), 581 (vs), 1429 (vs), 984 (vs), 1018 (vs), 939 (vs), 798 (vs), 1481 (vs), 633 (vs), 1632 (vs), 1167 (vs), 496 (vs), 527 (vs), 901 (vs), 461 (vs), 662 (vs), 1458 (vs), 474 (vs), 918 (s), 729 (s), 1398 (s), 1275 (s), 1306 (s), 708 (s), 1583 (s), 683 (m). **HRMS** (MALDI-TOF MS): m/z calcd. for $C_{34}H_{24}N_2O_2$ ($[M]^+$): 492.1832, found: 492.1832. **Elemental Analysis** (%): ($C_{34}H_{24}N_2O_2$) Calcd. C 82.91, H 4.91, N 5.69 found C 82.48, H 4.65, N 5.85. **UV/vis** (CH_2Cl_2) : λ_{max} (log(ε)) = 264 (4.72), 357 (3.89), 417 (3.93) nm.

Compound 3

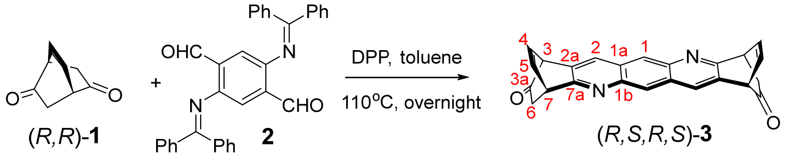

A solution of compound (*R*,*R*)-**1**[2,3] (27 mg, 0.2 mmol), diphenyl phosphate (DPP) (50 mg, 0.2 mmol) in toluene (5 mL) was heated in a two-neck flask at 110 °C. A solution of **2** (98.4 mg, 0.2 mmol) in toluene (5 mL) was transferred to the above solution using double-ended solvent transfer needle purged by compressed air over 3 hours. The reaction mixture was then heated at 110 °C overnight. After cooling to room temperature, the residue was collected by filtration, the orange solution was concentrated under a reduced pressure and purified by column chromatography on silica gel with a gradient of dichloromethane/ethyl acetate/ethanol from 10:10:1 to 4:4:1 (R_f = 0.53 for v/v/v = 4:4:1) as eluent to give compound (*R,S,R,S*)-**3** (20 mg, 27%) as colorless solid. **M.p.** 322 °C *dec*. **^{1}H NMR** (600 MHz, $CDCl_3$): δ = 8.55 ppm (s, 2H, H-1), 8.16 (s, 2H, H-2), 6.91 (t, J = 7.2 Hz, 2H, H-4), 6.73 (t, J = 7.8 Hz, 2H, H-5), 4.59 (dd, J = 6.0, 1.2 Hz, 2H, H-3), 4.49 (*br*, 2H, H-7), 2.50 (dd, J = 18.0, 3.0 Hz, 4H, H-6), 2.31 (dd, J = 18.0, 2.4 Hz, 2H, H-6). **^{13}C NMR** (151 MHz, $CDCl_3$): δ = 204.1 (C-7a), 164.3 (C-2a), 143.3 (C-1a), 136.9 (C-4), 131.6 (C-2), 131.2 (C-5), 128.4 (C-3a), 127.8 (C-1b), 126.9 (C-1), 58.3 (C-3), 43.9 (C-7), 36.1 (C-6). **FT-IR** (neat, ATR): $\tilde{\nu}$ = 3454 cm^{-1} (vs), 3902 (vs), 3101 (vs), 3096 (vs), 1867 (vs), 1475 (vs), 652 (vs), 1182 (vs), 837 (vs), 1013 (vs), 847 (vs), 3024 (vs), 1583 (vs), 808 (vs), 1225 (vs), 1354 (vs), 635 (vs), 789 (vs), 881 (vs), 1659 (vs), 1142 (vs), 955 (vs), 1290 (vs), 714 (vs), 1381 (vs), 868 (vs), 741 (s), 905 (s), 760 (s), 1092 (s), 922 (s). **MS** (APCI): $[M+H]^+$: *m/z* calcd. for ($C_{24}H_{17}N_2O_2^+$): 365.1285, found 365.1285. **Elemental Analysis** (%): ($C_{24}H_{16}O_2N_2 \cdot 0.5H_2O$) calcd. C 77.20, H 4.59 N 7.50 found C 76.93, H 4.68, N 7.47. **UV/vis** (CH_2Cl_2) : λ_{max} (log(ε)) = 265 (5.50), 340 (4.33), 349 (4.49), 356 (4.49), 366 (4.62) nm. **Emission** (CH_2Cl_2): λ_{max} (λ_{ex}) 382 (348), 402, 425 nm. The chiral HPLC trace (Chiralpak® IB, DCM/EA v/v 10/1) of (*R,S,R,S*)-**3**: retention time = 3.67 min. (−)-**3**: $[\alpha]_{20}^{578}$: = − 217.0, $[\alpha]_{20}^{546}$: = − 260.3, $[\alpha]_{20}^{436}$: = − 669.6. (+)-**3**: $[\alpha]_{20}^{578}$: = 221.6, $[\alpha]_{20}^{546}$: = 265.5, $[\alpha]_{20}^{436}$: = 696.9.

Compound 4

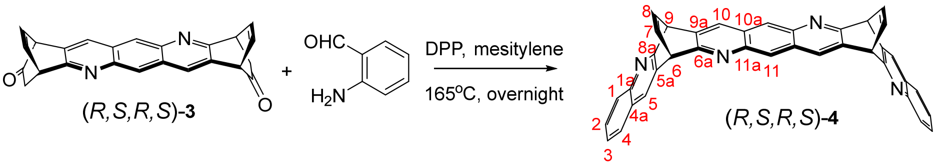

A solution of precursor (*R,S,R,S*)-**3** (15 mg, 0.04 mmol), 2-aminobenzaldehyde (23 mg, 0.19

mmol), diphenyl phosphate DPP (10 mg, 0.9 mmol) in mesitylene was heated in a round-bottom flask at 165 ℃ overnight. After cooling to room temperature, the black mixture was concentrated under a reduced pressure. Purification by column chromatography on silica gel with dichloromethane/ethyl acetate/ethanol = 4:4:1 (R_f = 0.26) as eluent gave compound (*R,S,R,S*)-**4** (20 mg, 91%) as colorless solid. **M.p.** 168 °C *dec.* **^{1}H NMR** (600 MHz, $CDCl_3$+TFA *d*): δ = 8.46 ppm (s, 2H, H-5), 8.42 (s, 2H, H-11), 8.40 (s, 2H, H-5), 8.20 (d, J = 8.4 Hz, 2H, H-4), 7.84 (d, J = 7.8 Hz, 2H, H-1), 7.76 (td, J = 7.8, 1.2 Hz, 2H, H-3), 7.60 (t, J = 7.8 Hz, 2H, H-2), 7.24 (td, J = 7.2, 1.2 Hz, 2H, H-8), 7.19 (td, J = 7.2, 1.8 Hz, 2H, H-7), 6.29 (d, J = 6.0 Hz, 2H, H-6), 5.86 (d, J = 6.0 Hz, 2H, H-9). **^{13}C NMR** (151 MHz, $CDCl_3$+TFA *d*): δ = 163.8 (C-9a), 161.4 (C-5a), 140.6 (C-11a), 138.4 (C-8), 138.1 (C-4a), 137.1 (C-7), 136.4 (C-5), 134.6 (C-8a), 133.1 (C-10), 133.0 (C-3), 131.6 (C-6a), 129.4 (C-2), 128.4 (C-1), 127.6 (C-10a), 126.6 (C-1a), 126.0 (C-11), 123.2 (C-4), 49.7 (C-6), 47.0 (C-9). **FT-IR** (neat, ATR): $\tilde{\nu}$ = 2341 cm^{-1} (vs), 3751 (vs), 3518 (vs), 1794 (vs), 1998 (vs), 3314 (vs), 621 (vs), 3124 (vs), 1877 (vs), 2557 (vs), 777 (vs), 3950 (vs), 2253 (vs), 1896 (vs), 1514 (vs), 970 (vs), 1420 (vs), 2808 (vs), 3622 (vs), 3846 (vs), 1663 (vs), 3011 (vs), 1583 (vs), 1286 (vs), 1063 (vs), 795 (vs), 1560 (vs), 1026 (vs), 1387 (vs), 1157 (vs), 1200 (vs), 1109 (vs), 829 (s), 1082 (s), 1140 (s), 889 (s), 845 (s), 943 (s), 872 (s), 1180 (s), 712 (s), 912 (s). **MS** (MALDI-TOF MS): $[M+H]^+$: *m/z* calcd. for ($C_{28}H_{23}N_4^+$): 535.1917, found 535.1918. **Elemental Analysis** (%): ($C_{38}H_{22}N_4{\cdot}2H_2O$) calcd. C 79.98, H 4.59, N 9.82 found C 80.72, H 4.58, N 9.44. **UV/vis** (CH_2Cl_2): λ_{max} (log(ε)) = 276 (4.62), 322 (3.97), 355 (4.02), 373 (4.22) nm. **Emission** (CH_2Cl_2): λ_{max} (λ_{ex}) 377 (345), 397 nm. The chiral HPLC trace (Chiralpak® IB, DCM/EA/IPA v/v/v 12/12/1) of (*R,S,R,S*)-**4**: retention time = 4.28min. (+)-**4**: $[\alpha]_{20}^{578}$: = 186.0, $[\alpha]_{20}^{546}$: = 221.1, $[\alpha]_{20}^{436}$: = 409.6. (−)-**4**: $[\alpha]_{20}^{578}$: = − 197.1, $[\alpha]_{20}^{546}$: = − 232.7, $[\alpha]_{20}^{436}$: = − 432.8.

2. NMR Spectra

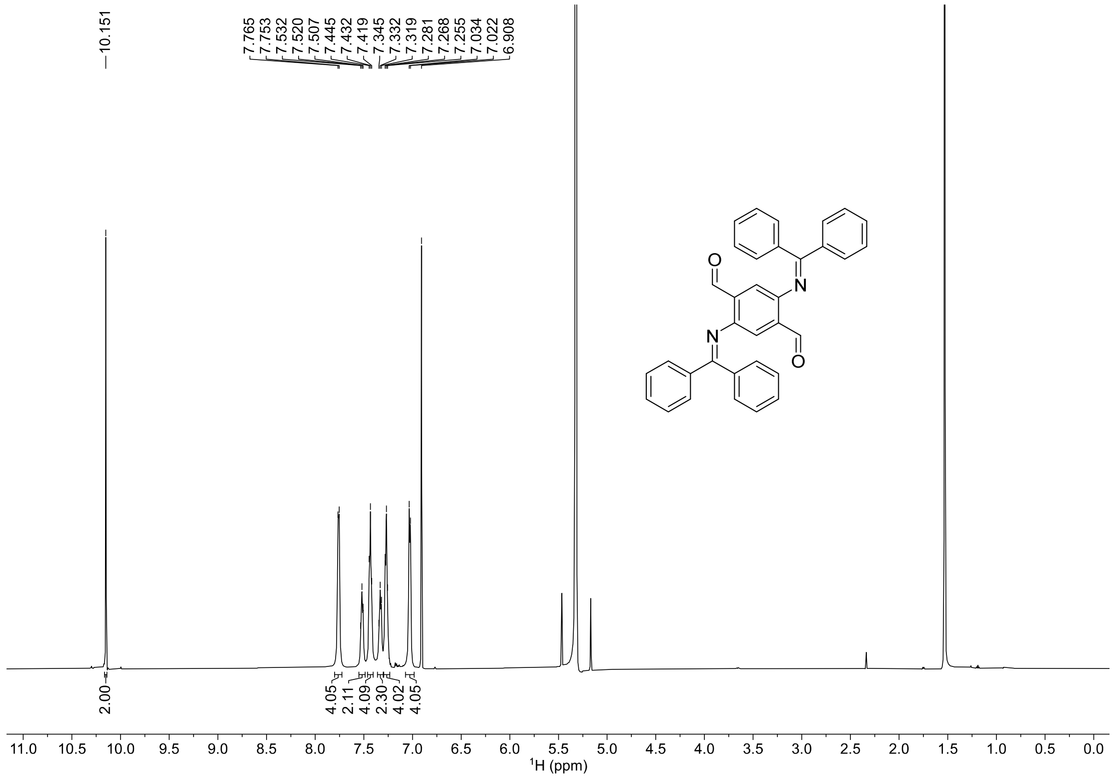

Figure S1. ^{1}H NMR spectrum of **2** (CD_2Cl_2, 600MHz).

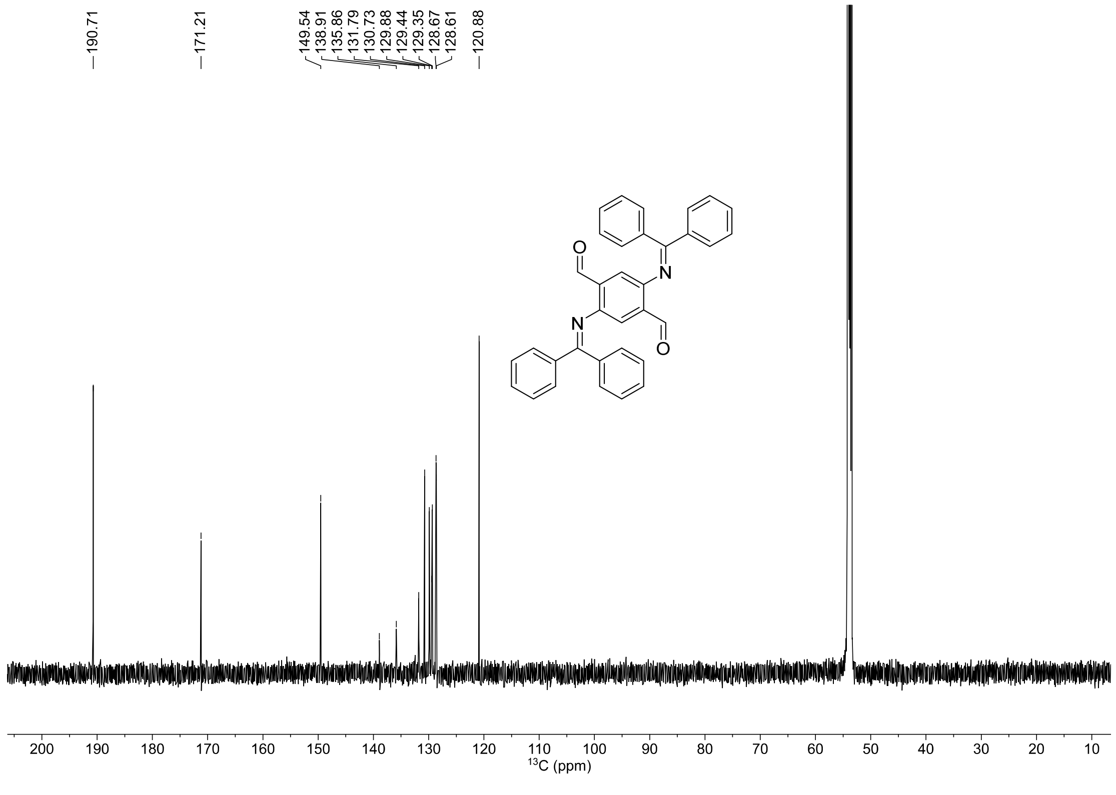

Figure S2. ^{13}C NMR spectrum of **2** (CD_2Cl_2, 151MHz).

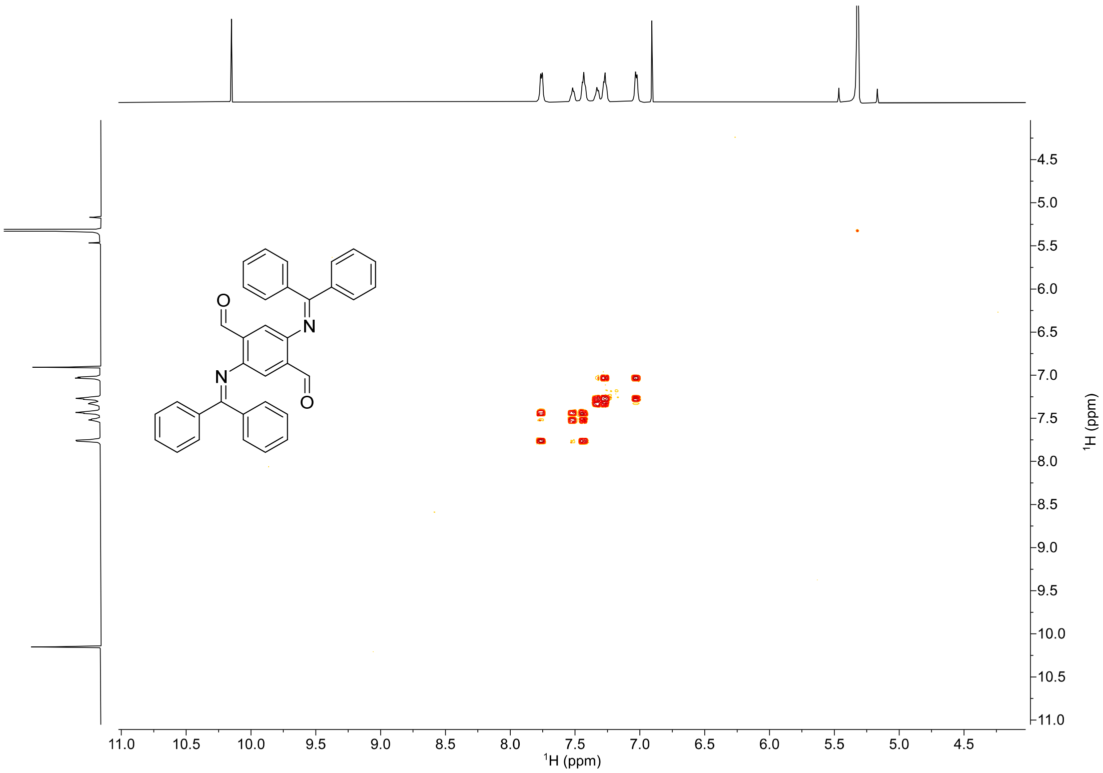

Figure S3. ^{1}H-^{1}H COSY NMR spectrum of **2** (CD$_2$Cl$_2$, 600 MHz, 600 MHz).

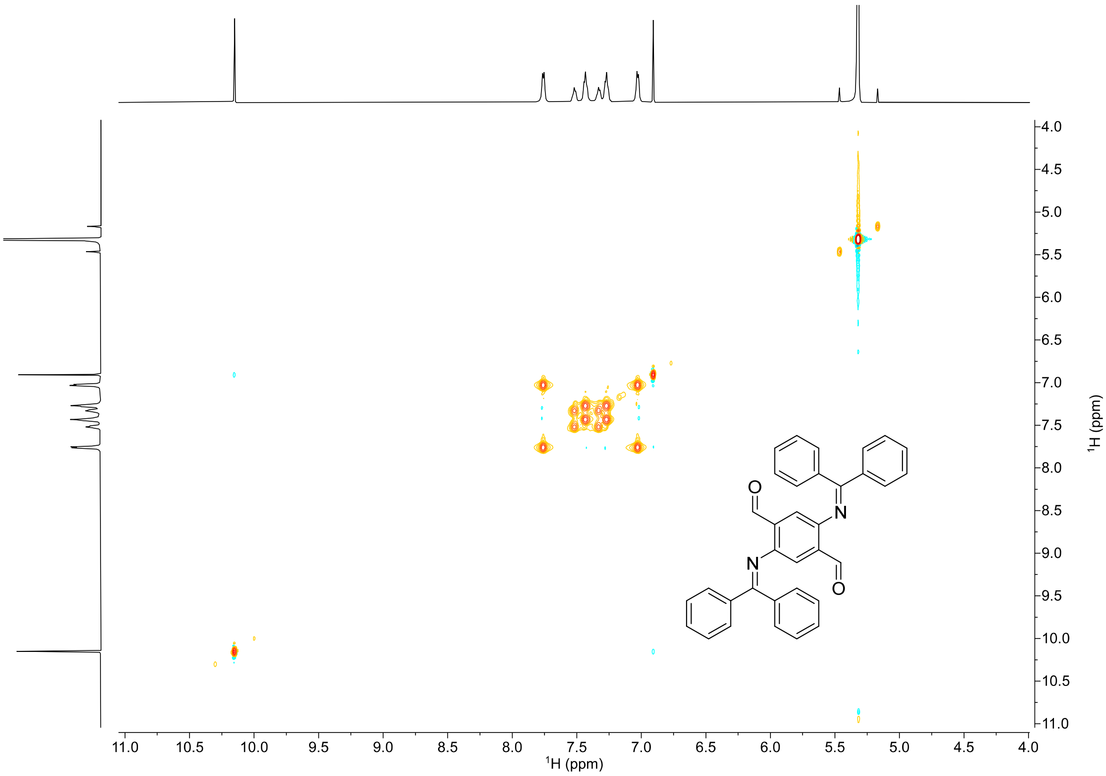

Figure S4. ^{1}H-^{1}H NOESY NMR spectrum of **2** (CD$_2$Cl$_2$, 600 MHz, 600 MHz).

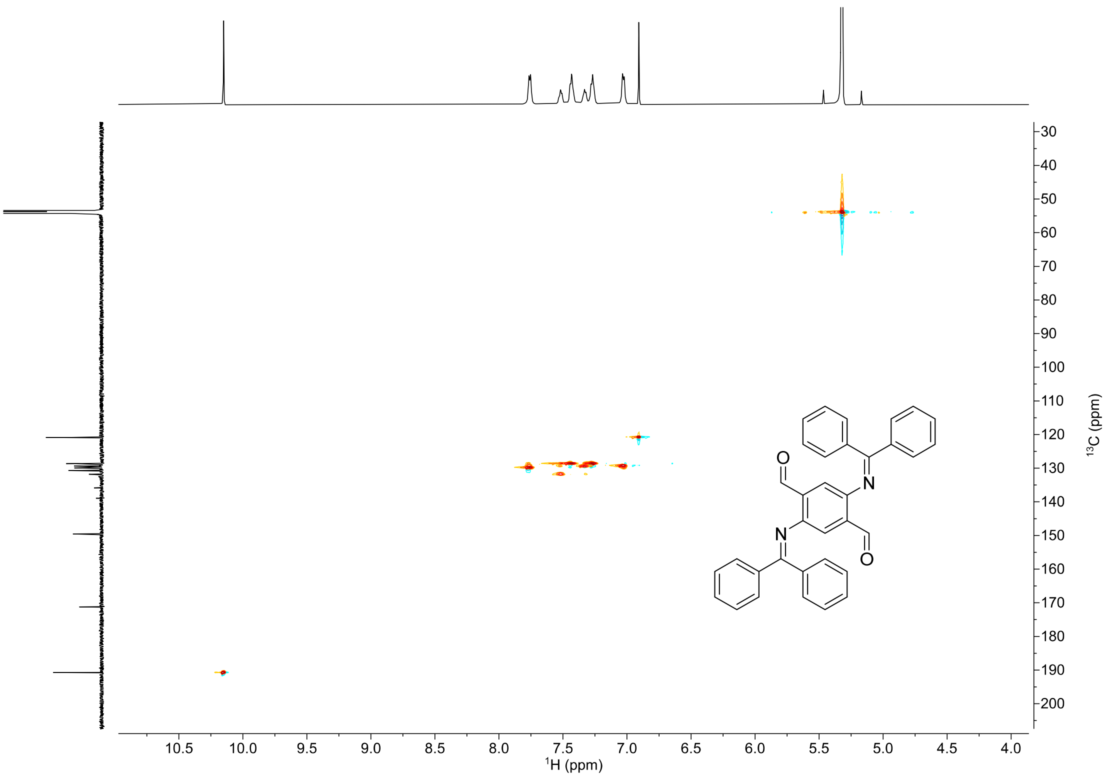

Figure S5. ^{1}H-^{13}C HSQC-EDITED NMR spectrum of **2** (CD_2Cl_2, 600 MHz, 151 MHz).

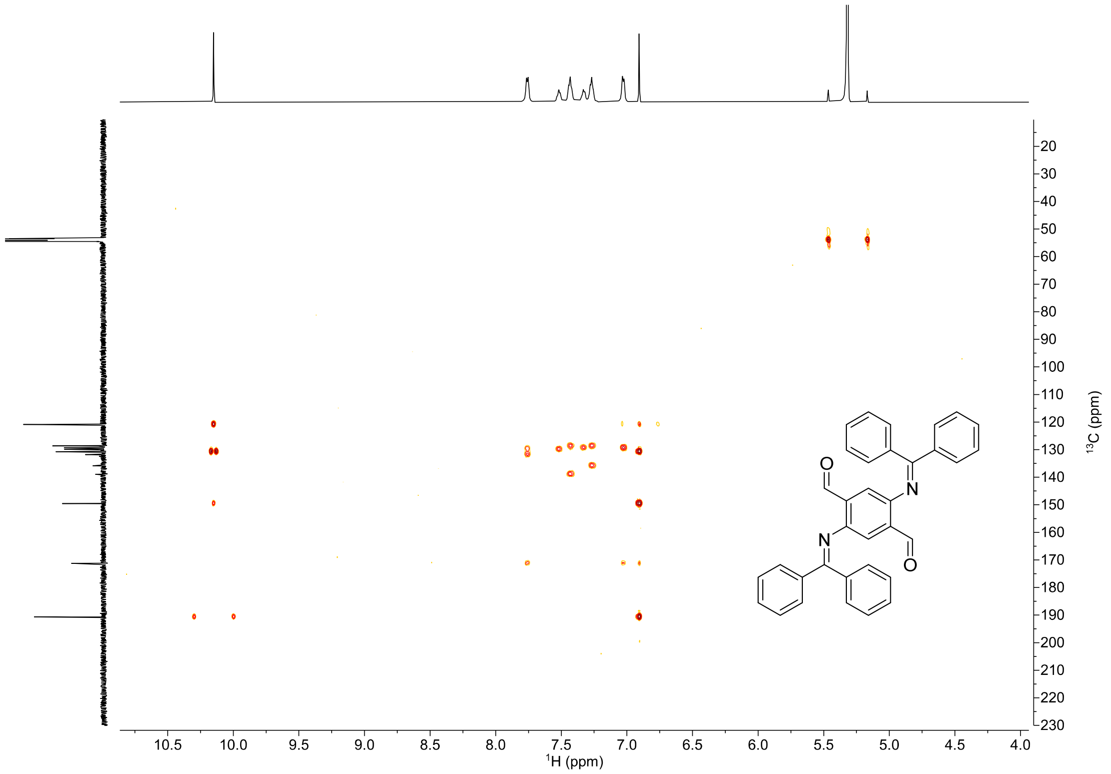

Figure S6. ^{1}H-^{13}C HMBC NMR spectrum of **2** (CD_2Cl_2, 600 MHz, 151 MHz).

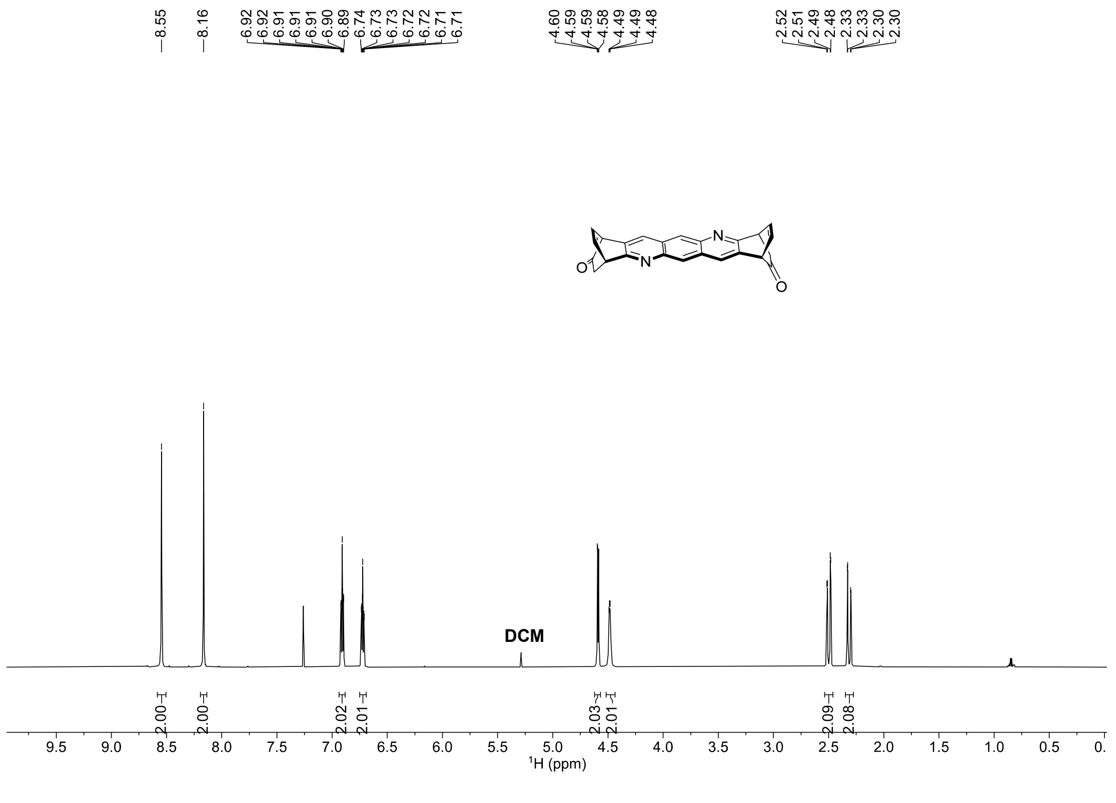

Figure S7. ^{1}H NMR spectrum of **3** (CDCl$_3$, 600MHz).

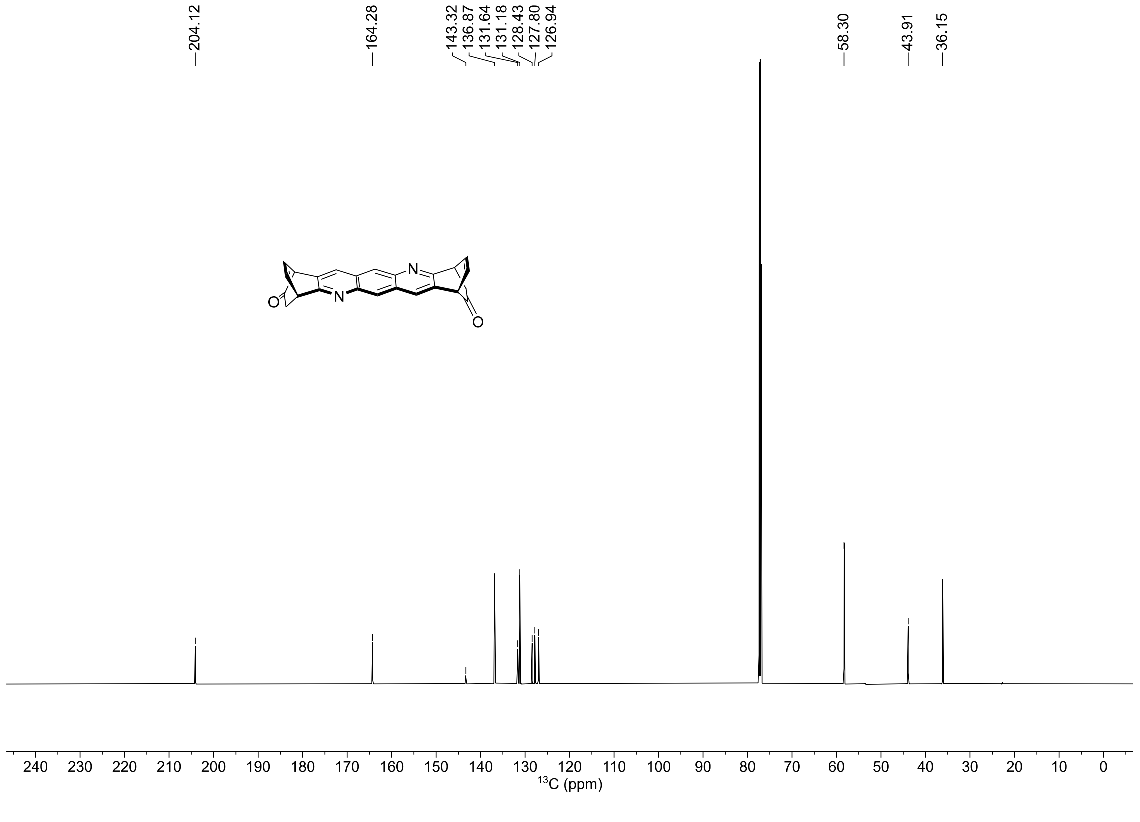

Figure S8. ^{13}C NMR spectrum of **3** (CDCl$_3$, 151MHz).

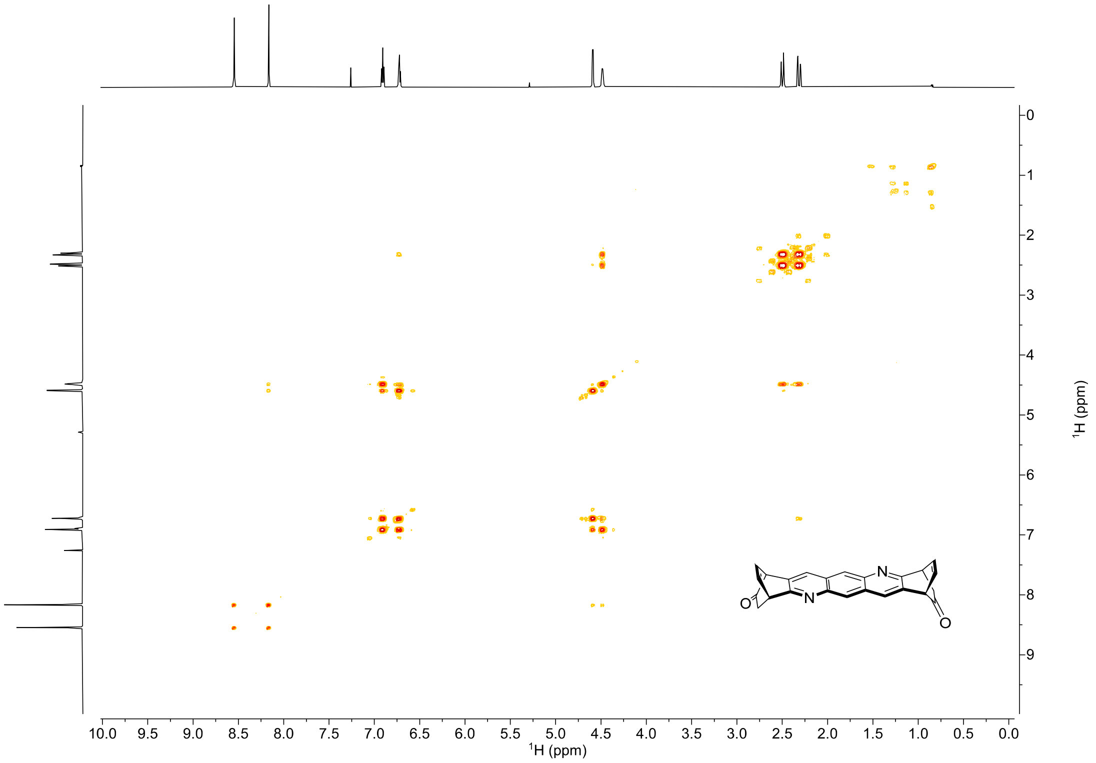

Figure S9. ^{1}H-^{1}H COSY NMR spectrum of **3** (CDCl3, 600 MHz, 600 MHz).

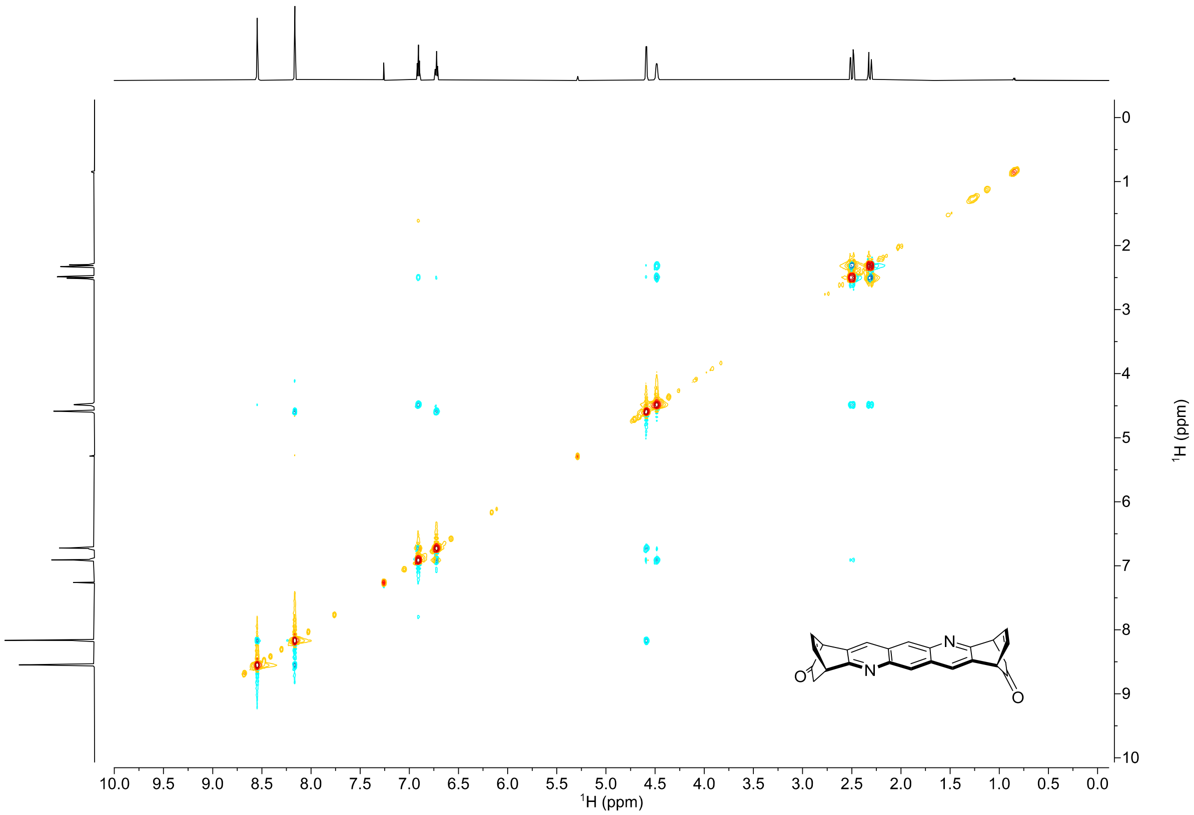

Figure S10. ^{1}H-^{1}H NOESY NMR spectrum of **3** (CDCl3, 600 MHz, 600 MHz).

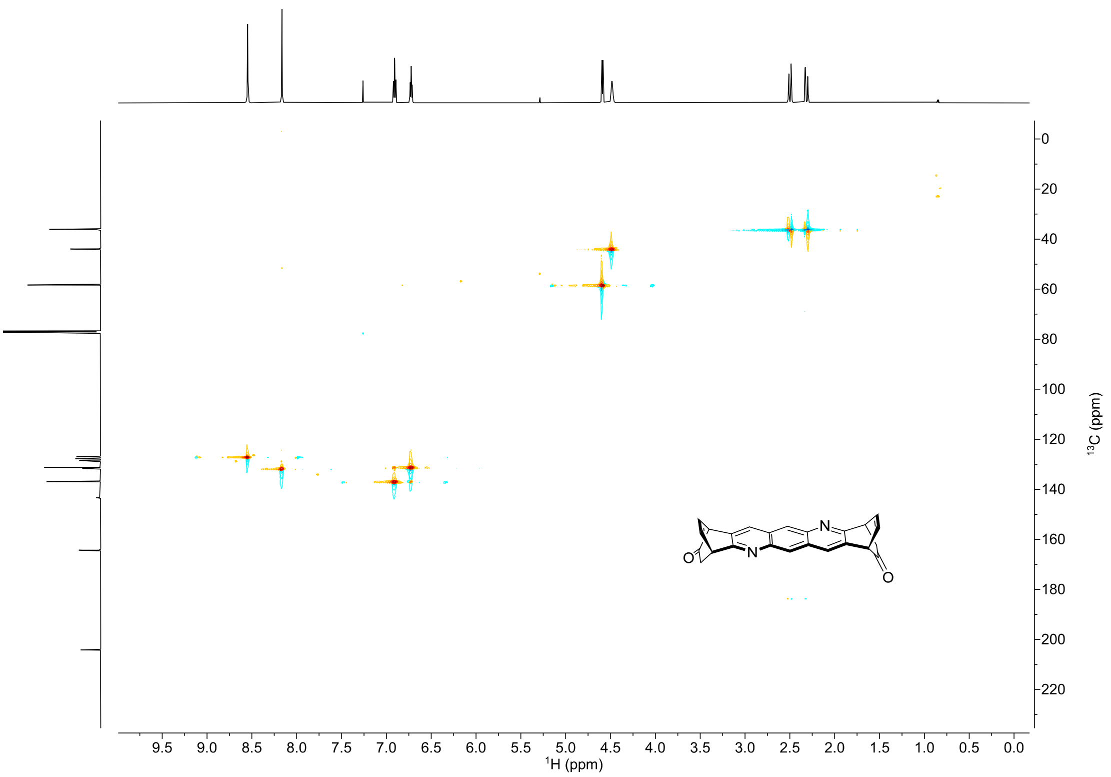

Figure S11. ^{1}H-^{13}C HSQC-EDITED NMR spectrum of **3** ($CDCl_3$, 600 MHz, 151 MHz).

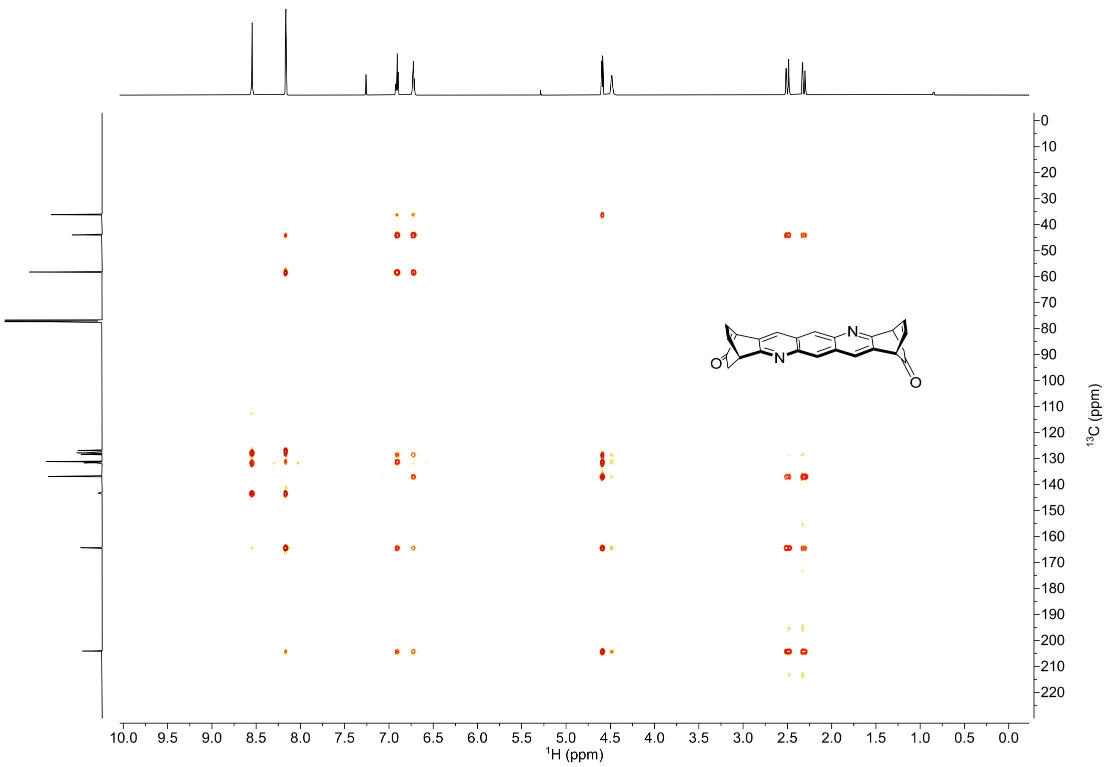

Figure S12. ^{1}H-^{13}C HMBC NMR spectrum of **3** ($CDCl_3$, 600 MHz, 151 MHz).

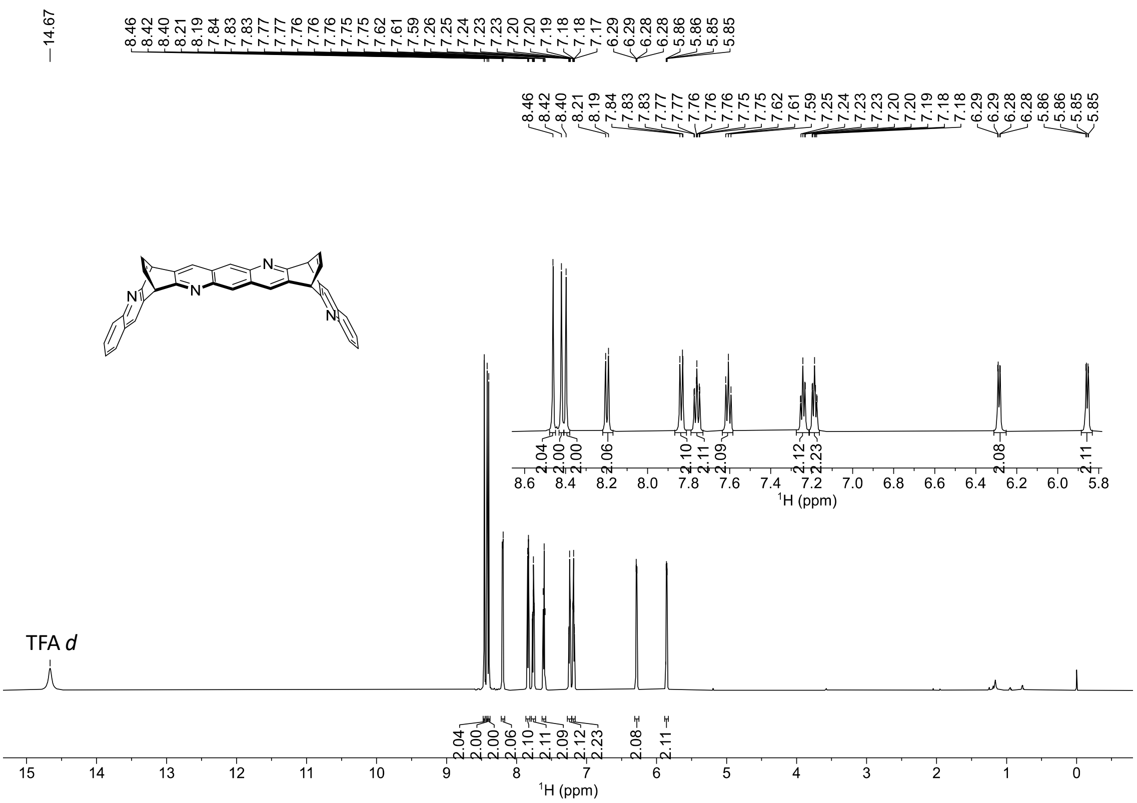

Figure S13. ^{1}H NMR spectrum of **4** ($CDCl_3$+TFA *d*, 600MHz).

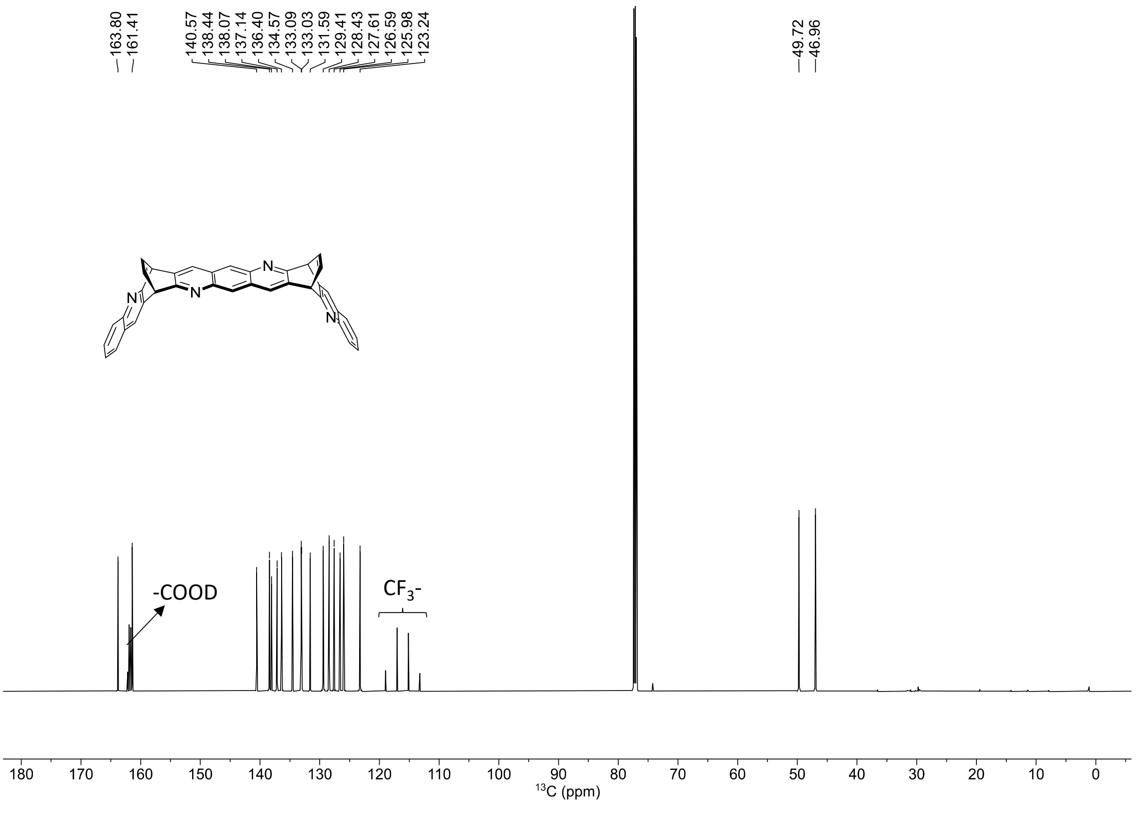

Figure S14. ^{13}C NMR spectrum of **4** ($CDCl_3$+TFA *d*, 151 MHz).

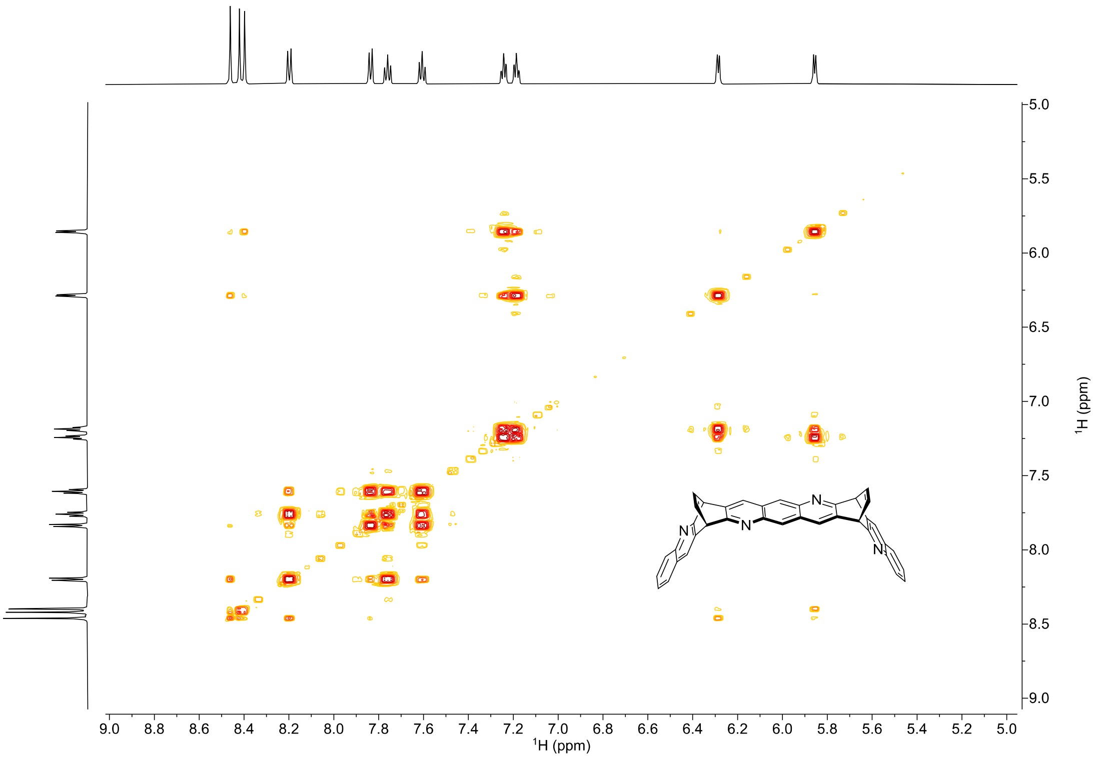

Figure S15. ^{1}H-^{1}H COSY NMR spectrum of **4** (CDCl3+TFA *d*, 600 MHz, 600 MHz).

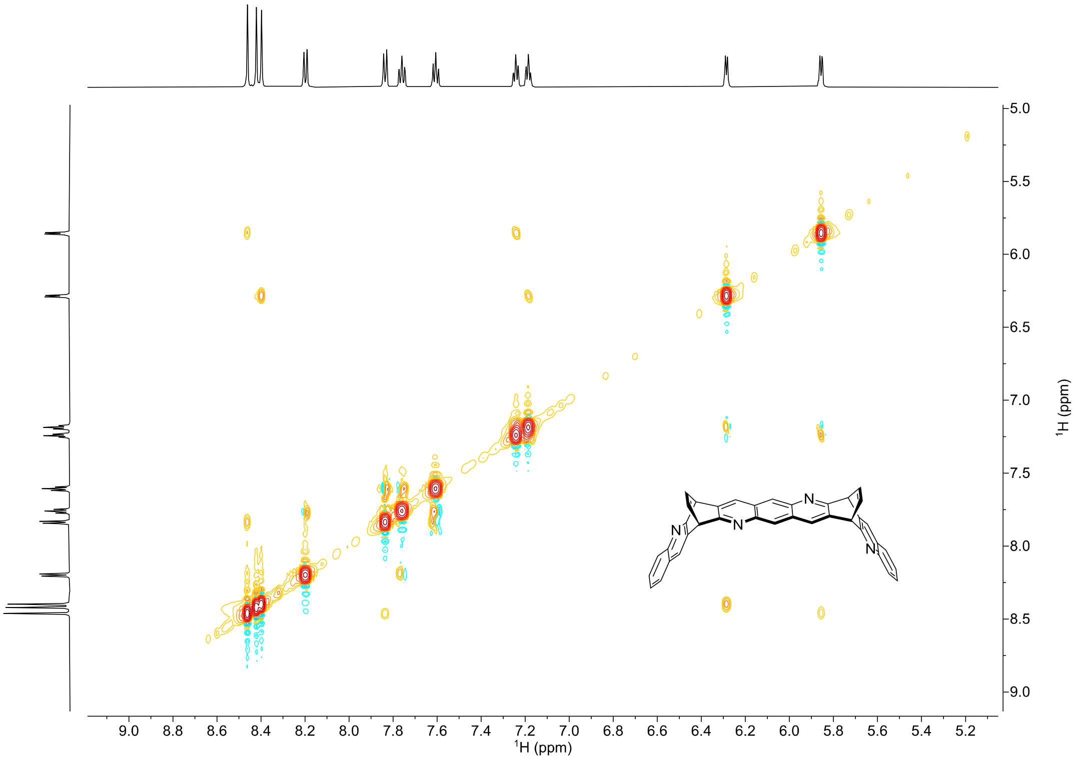

Figure S16. ^{1}H-^{1}H NOESY NMR spectrum of **4** (CDCl3+TFA *d*, 600MHz, 600MHz).

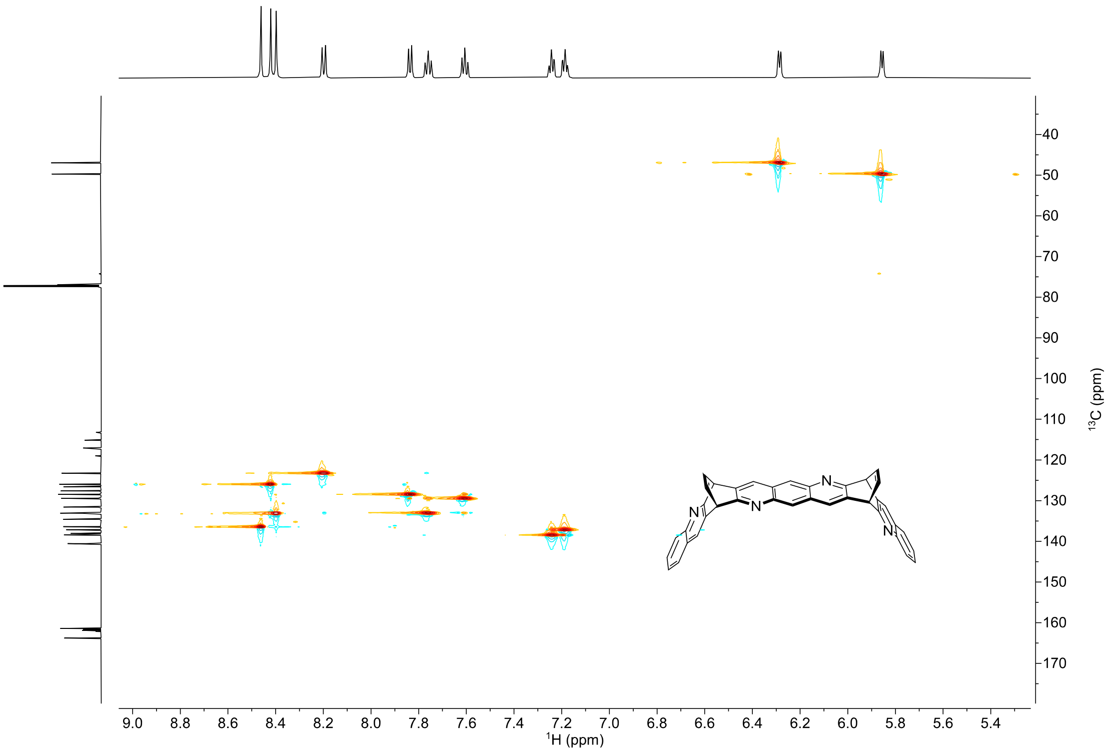

Figure S17. ^{1}H-^{13}C HSQC-EDITED NMR spectrum of **4** ($CDCl_3$+TFA *d*, 600 MHz, 151 MHz).

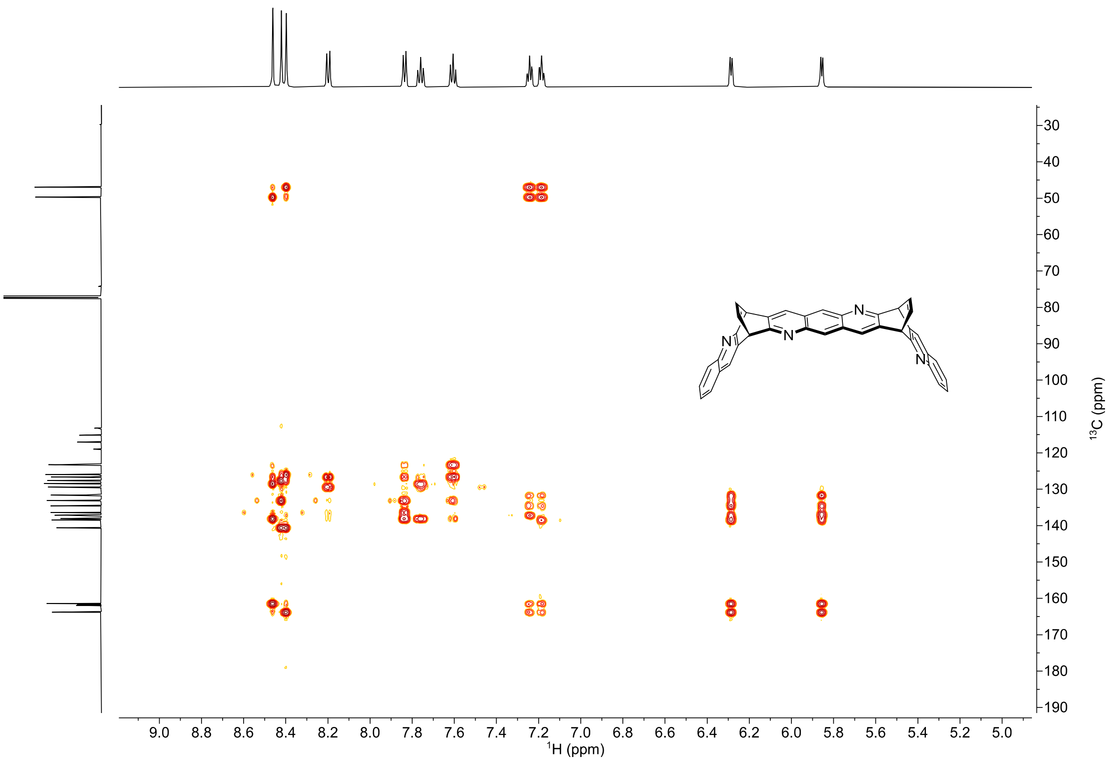

Figure S18. ^{1}H-^{13}C HMBC NMR spectrum of **4** ($CDCl_3$+TFA *d*, 600 MHz, 151 MHz).

3. HR-MS spectra

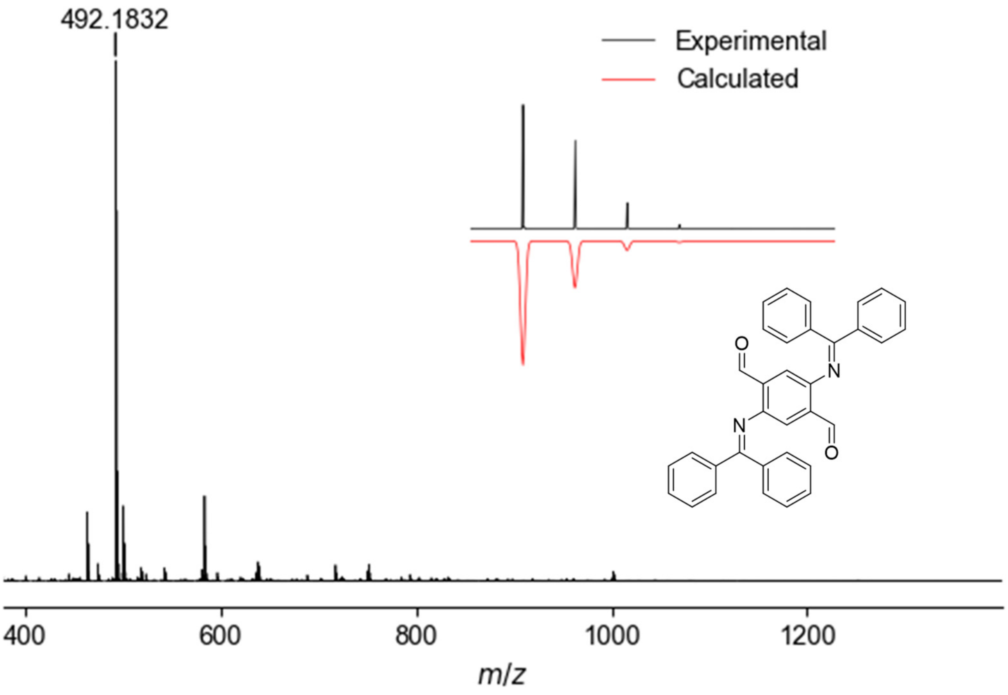

Figure S19. High resolution mass spectra (MALDI-TOF) of **2**.

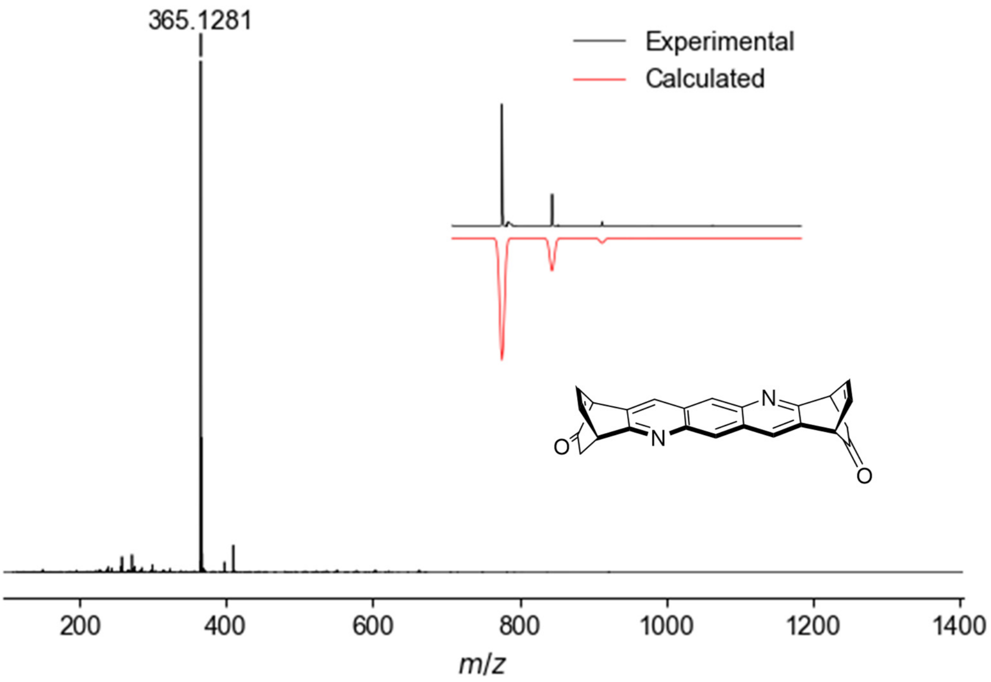

Figure S20. High resolution mass spectra (APCI) of **3**.

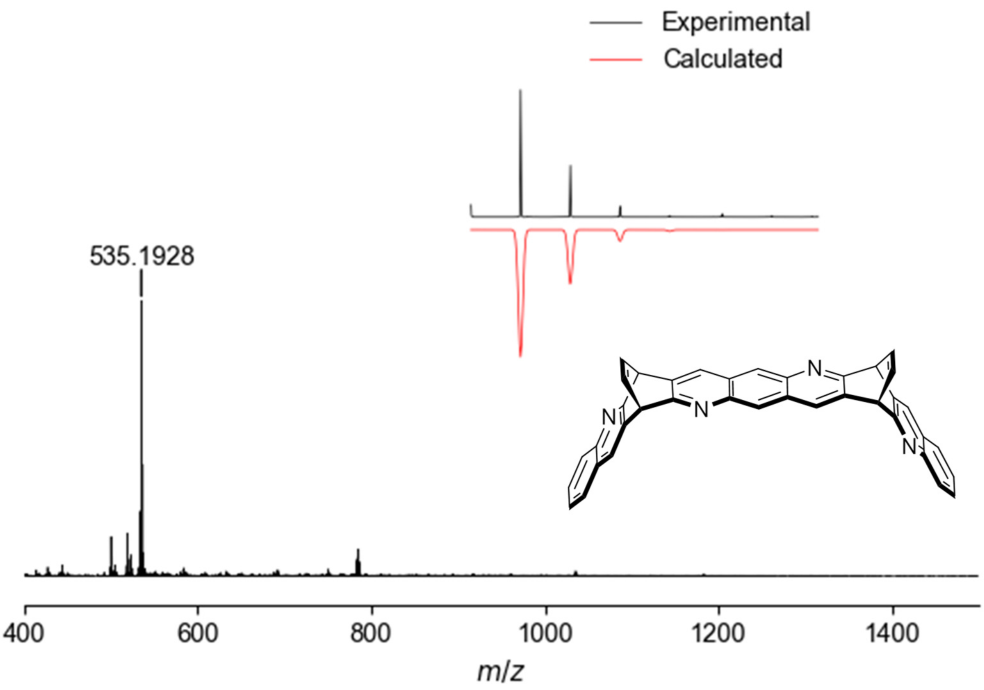

Figure S21. High resolution mass spectra (MALDI-TOF) of **4**.

4. IR spectra

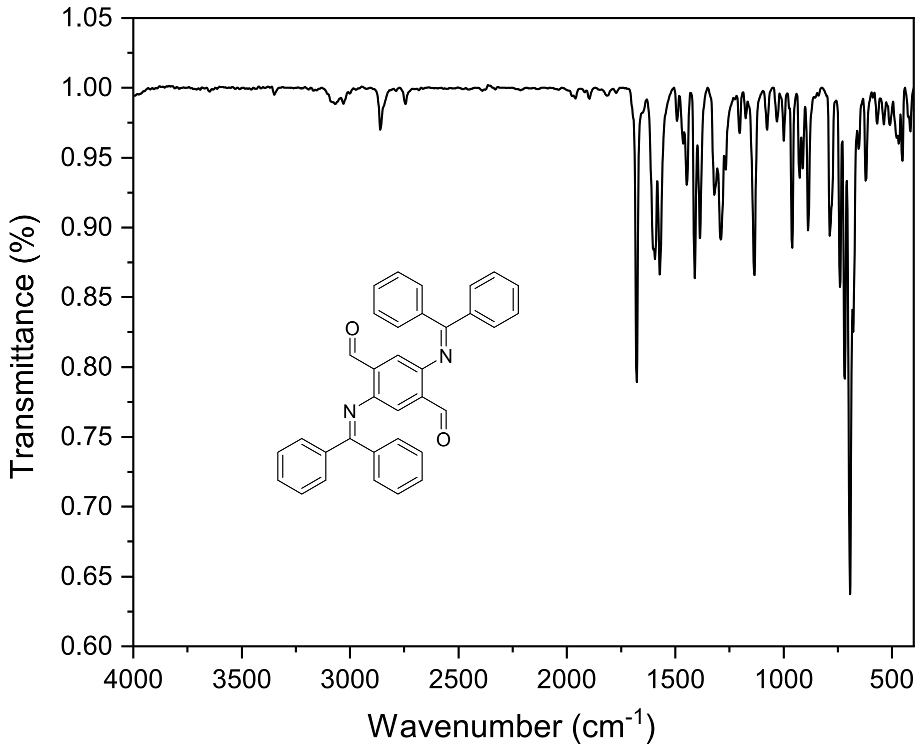

Figure S22. IR spectrum (ATR) of **2**.

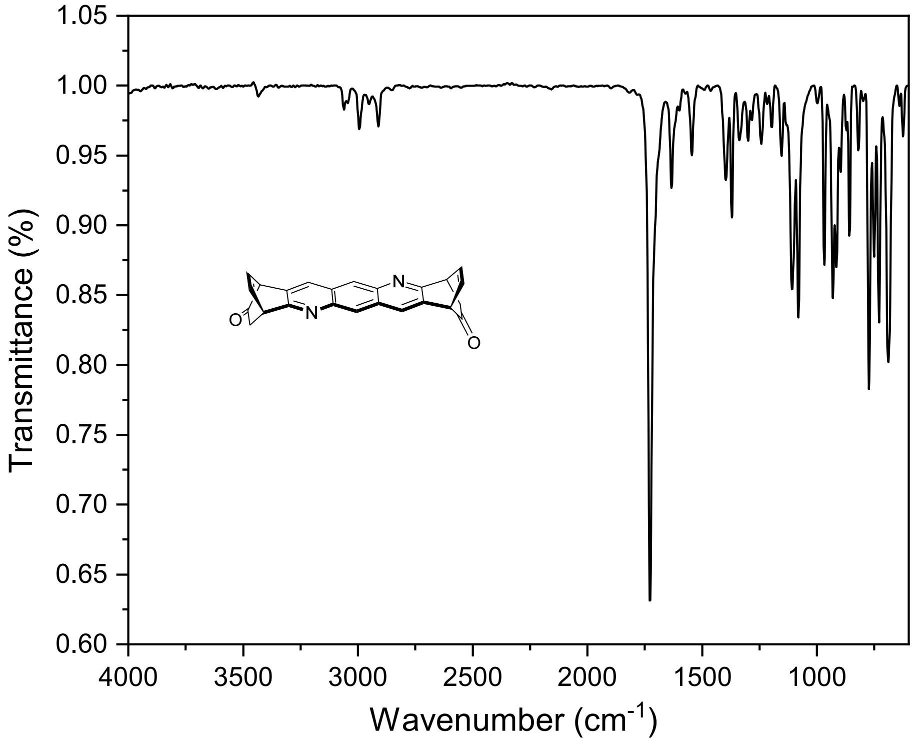

Figure S23. IR spectrum (ATR) of **3**.

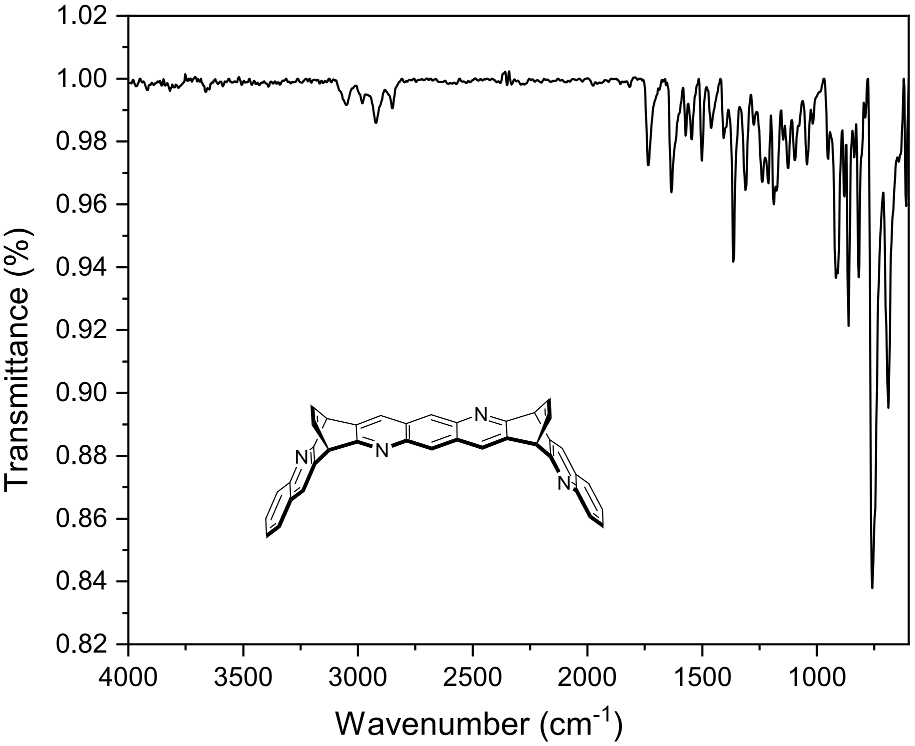

Figure S24. IR spectrum (ATR) of **4**.

5. UV-vis and fluorescence spectra

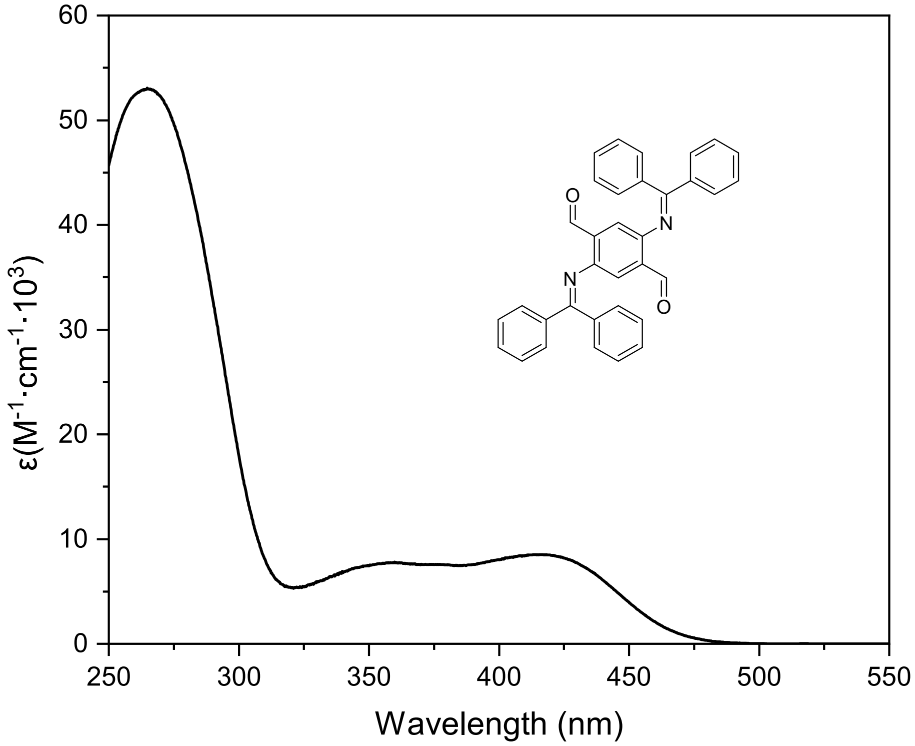

Figure S25. UV-vis absorption spectrum of **2** in CH_2Cl_2.

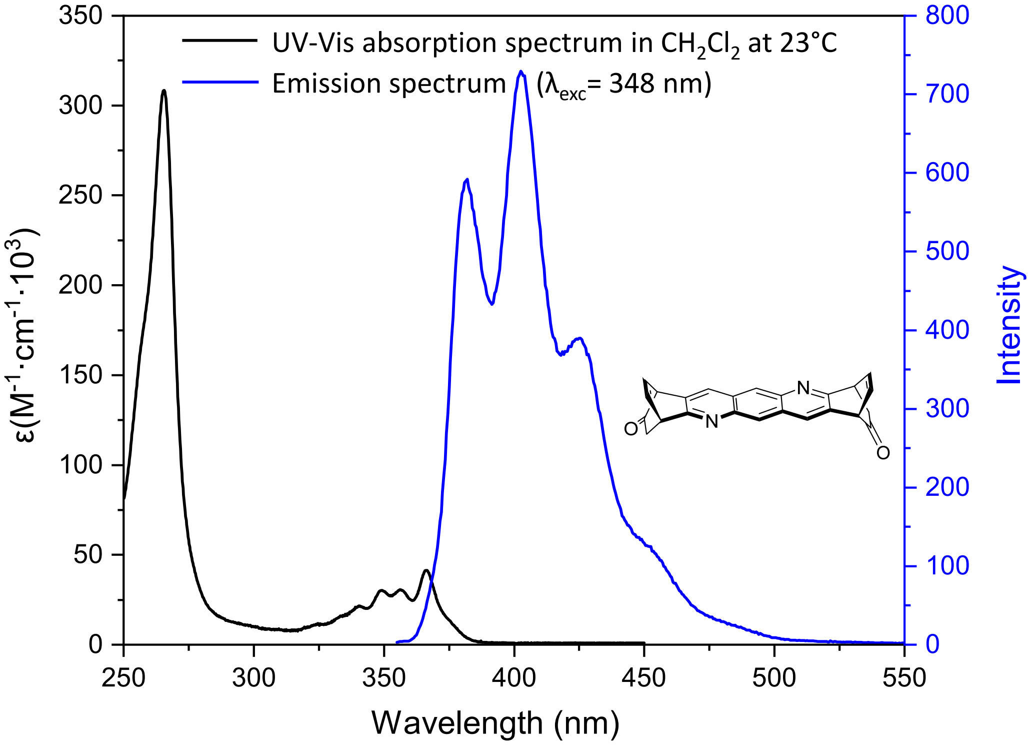

Figure S26. UV-vis absorption and fluorescence spectra of **3**.

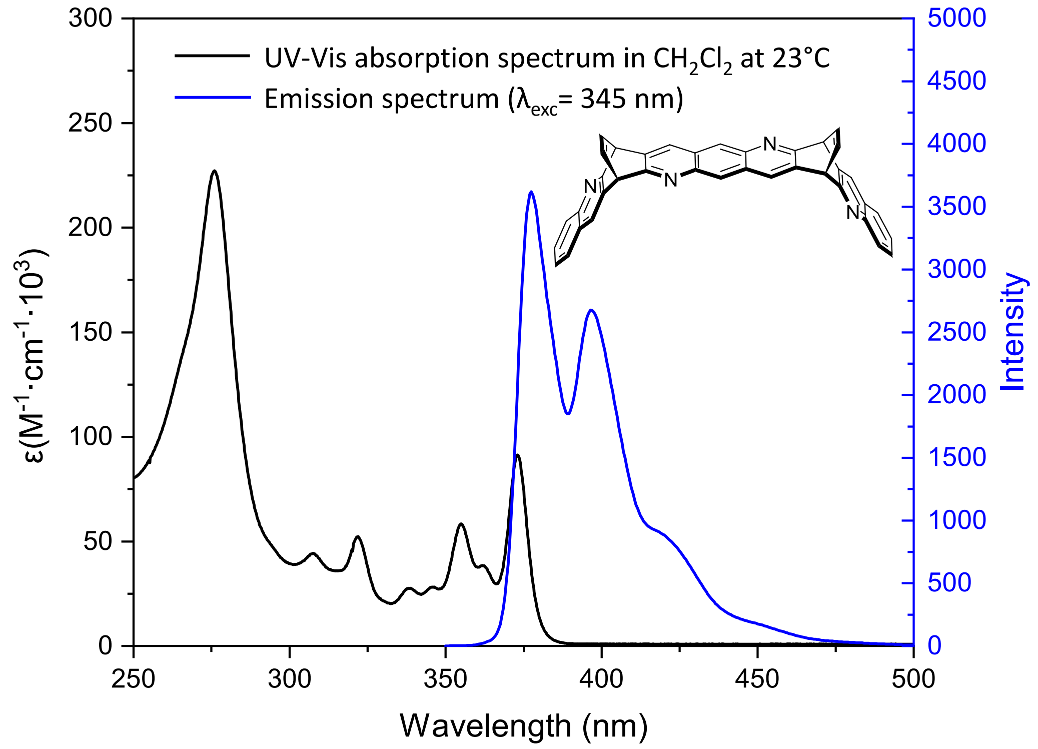

Figure S27. UV-vis absorption and fluorescence spectra of **4**.

6. HPLC Chromatograms

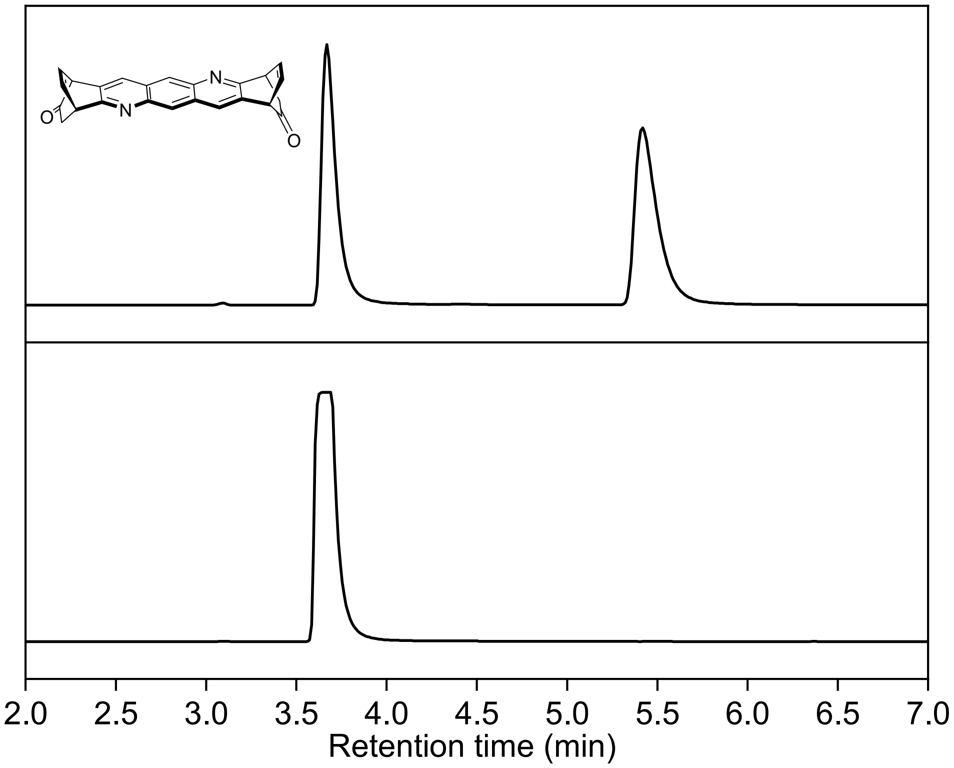

Figure S28. Analytical HPLC traces of the racemic mixture of **3** and (*R,S,R,S*)-**3** (IB column, DCM /EA 10:1, 1 mL/min, 254 nm).

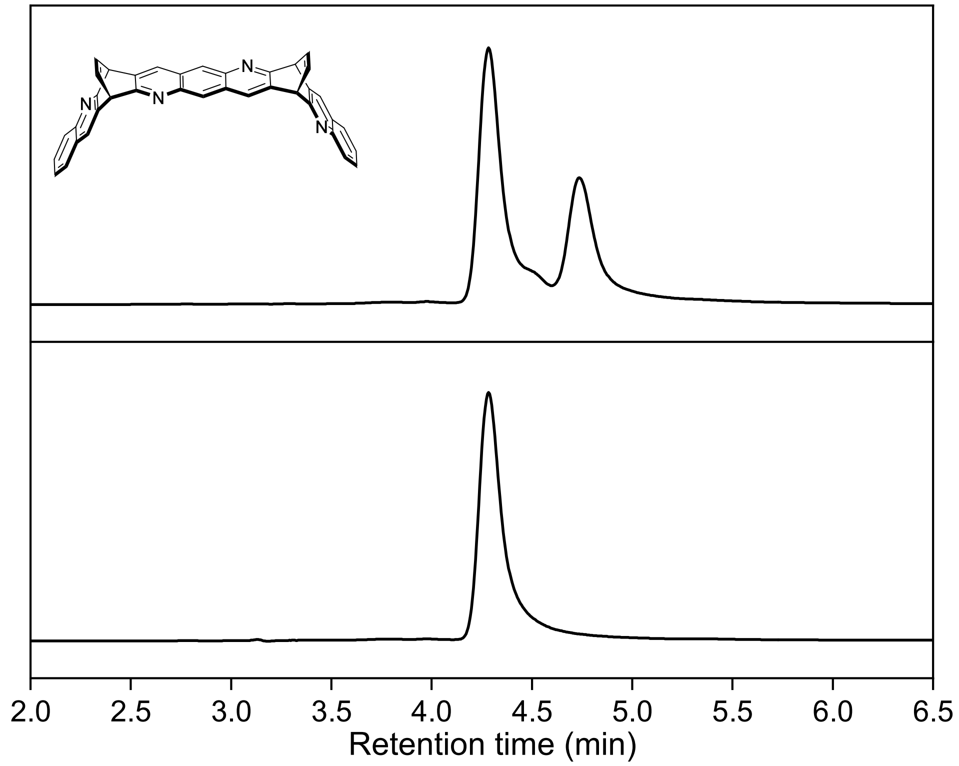

Figure S29. Analytical HPLC traces of the racemic mixture of **4** and (*R,S,R,S*)-**4** (IB column, DCM/EA/IPA 12:12:1, 1 mL/min, 272 nm).

7. CD spectra

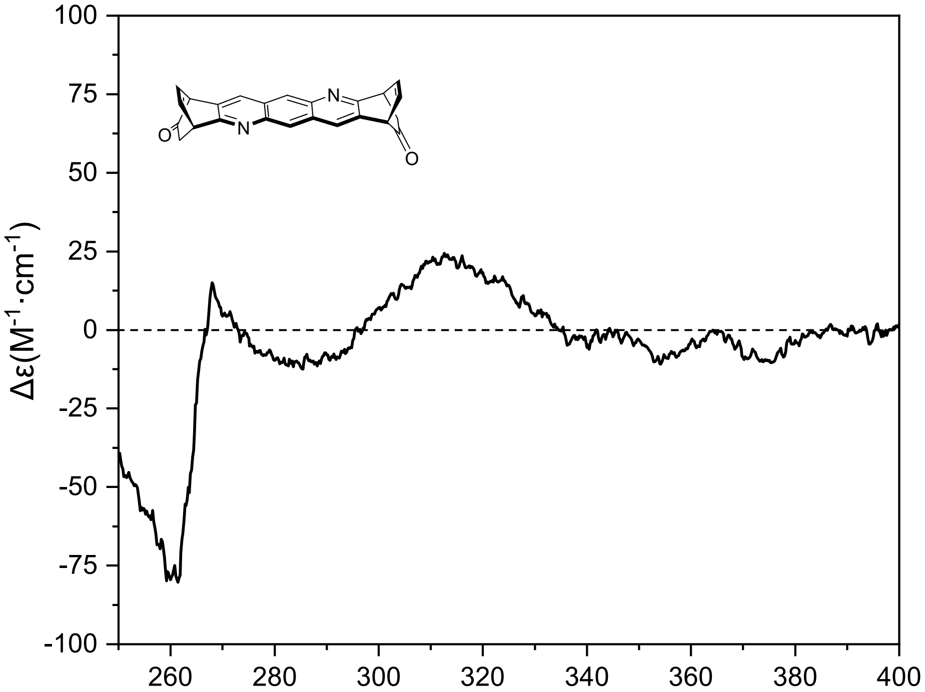

Figure S30. CD spectrum of the (*R,S,R,S*)-**3** in DCM, 25°C.

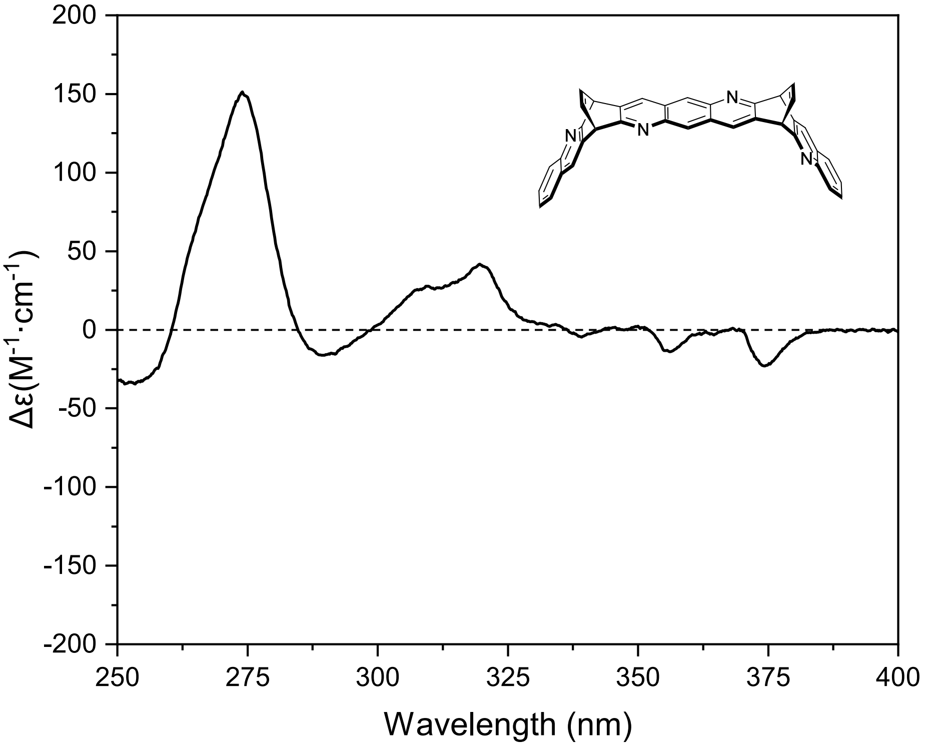

Figure S31. CD spectrum of the (*R,S,R,S*)-**4** in DCM, 25°C.

8. Experimental and Theoretical Details and Additional Data

On-Surface Synthesis

Sample Preparation

The atomically clean Au(111) (MaTecK) surface was obtained by cycles of argon-ion sputtering and annealing at 800 K. The trietheno-bridged precursor was vapor-deposited from a standard Knudsen cell heated to 520 K onto the Au(111) substrates held at room temperature. Carbon monoxide (CO) molecules were dosed onto the sample surfaces for tip modification.

Scanning Probe Microscopy Characterization

Experiments were performed by using an ultrahigh vacuum low-temperature scanning tunneling microscope (UHV LT-STM, Scienta Omicron) with a base pressure better than 1×10^{-10} mbar. All STM images were acquired in constant-current or constant-height mode by using an electrochemically etched tungsten tip at 4.2 K. All given voltages were applied to the sample with respect to the tip. Nc-AFM measurements were performed at 4.2 K with tungsten tips placed on a qPlus tuning fork sensor[45] driven at its resonance frequency (28,650 Hz) with a constant amplitude of ∼70 pm. The tips were functionalized with a single CO molecule at the tip apex picked up from the Au surface after dosing with CO.[36] The Δz was positive (negative) when the tip–surface distance was increased (decreased) with respect to the STM set point at which the feedback loop was opened. The dI/dV spectra were recorded by using a lock-in amplifier with a modulation frequency of 579 Hz and an amplitude of 15-20 mV. dI/dV maps were collected in constant-current or constant-height mode as specified in the captions.

Computational methods

The gas-phase electronic structure, local density of states (LDOS) maps and aromaticity calculations were performed with the Gaussian software package. The B3LYP functional was used in the spin-restricted and unrestricted formalism. The 6-311G** basis set was used for geometry optimizations, while single point and aromatic properties were calculated using the 6-311+G** basis set. The radical characters were calculated based on the natural orbital occupation numbers with the unrestricted Hartree Fock (UHF) method using Yamaguchi's spin projection scheme[46]. The $NICS_{zz}(1)$[37,47] was calculated with the GIAO-B3LYP method as the

negative of the magnetic shielding tensor component perpendicular to each local cycle evaluated at height 1 Å away from the center. The anisotropy of the induced current density (ACID)[43] calculation was performed with the CSGT method.[48] The magnetically induced current density susceptibility was calculated using the gauge-including magnetically induced current (GIMIC) method.[44] The magnetically induced current density plots were generated with the ParaView program.[49] The calculation results were analyzed with the help of Multiwfn package[50] and visualized by Visual Molecular Dynamics.[51] GIMIC uses basis set information, the atomic orbital density matrix, and the magnetically perturbed atomic orbital density matrices as input data, which were obtained by performing nuclear magnetic resonance shielding calculations.

Multiconfigurational quantum chemistry calculations were done with the CASSCF method with the def2-TZVP basis set using ORCA 6.0.1 packages.[52] For both tetraazanonacene and nonacene, the relaxed geometry from DFT was employed for unrestricted Hartree Fock calculation. The resulting natural orbitals were used as input for the CASSCF calculation. A complete active space of 14 electrons correlated within 14 orbitals was selected, that is, CASSCF(14,14), a limited size of the reference space was found proper to describe the acenes with similar size.

Single-point state-specific DMRG-CASSCF calculations employing the def2-TZVP basis set were performed on the above mentioned DFT optimized geometries with the pySCF package[53] interfaced with Block2 library,[54] using unrestricted Hartree-Fock natural orbital as initial guess. The number of renormalized states m was set to 1000, which is sufficient to capture the multiconfigurational character effectively without significant truncation error.

8.1 Surface with Low Coverage of Precursor Molecules 4.

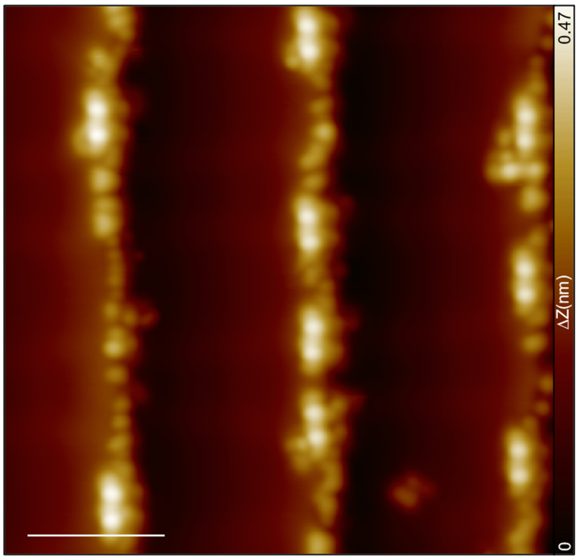

Figure S32. Large-scale STM image of surface with low coverage of the precursor molecule **4**. At low coverage, the precursor molecules show preferred adsorption at the step edges. Scanning parameters: $V_s = 0.2$ V, $I_t = 20$ pA. Scale bars: 5 nm.

8.2 Attempted Thermal Deprotection of the Intact Precursor by Annealing

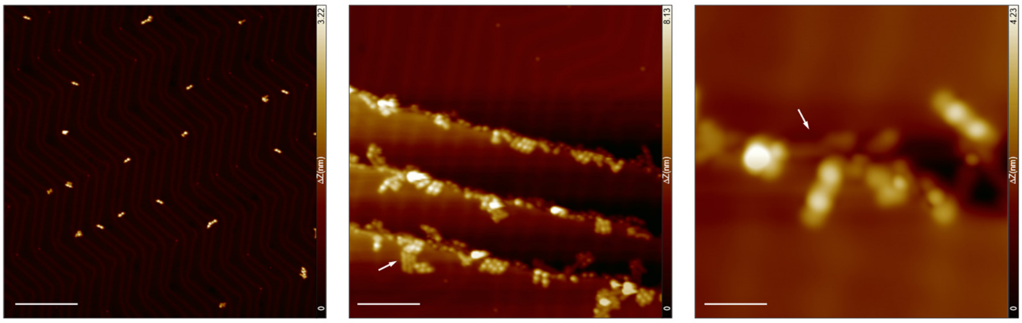

Figure S33. STM images taken after annealing the sample to 500 K. The white arrow indicates the rarely observed flattened molecules. Scanning parameters: $V_s = 0.15$ V, $I_t = 30$ pA. Scale bars: (left to right) 20 nm, 9 nm and 2 nm, respectively.

8.3 Additional High-Resolution Images of the Intact Precursor 4 and Tetraazanonacene.

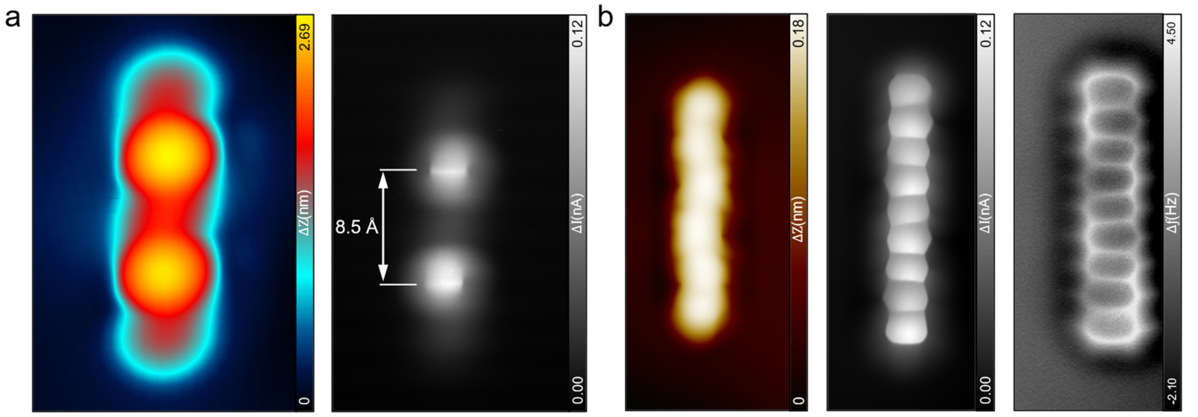

Figure S34. High-resolution images of the precursor molecule 4 and tetraazanonacene. (a) STM and bond-resolved-STM (BR-STM) image of the intact precursor. (b) (left to right) STM, BR-STM and midium tip height nc-AFM images of tetraazanonacene. Scanning parameters: (a) STM, $V_s = 0.15$ V, $I_t = 20$ pA; BR-STM, $V_s = 10$ mV. (b) STM, $V_s = 0.1$ V, $I_t = 50$ pA; BR-STM and nc-AFM, $V_s = 10$ mV. Scale bars: (a) 2×3 nm; (b) STM, 1.7×3.7 nm; BR-STM and nc-AFM, 3.2×1.2 nm.

8.4 High Mobility of Tetraazanonacene on Au(111).

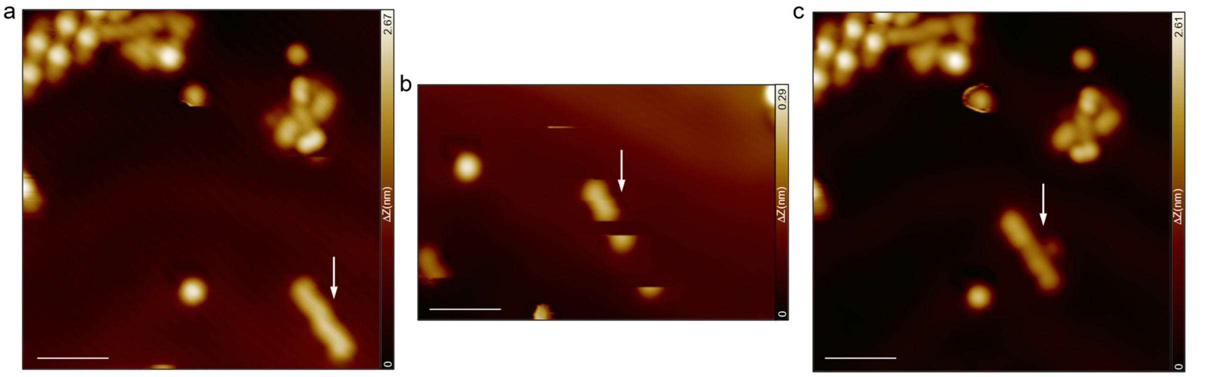

Figure S35. Large-scale STM images of tetraazanonacene during continuous scanning. (a-c) STM images showing the lateral diffusion of tetraazanonacene during scanning, indicating rather weak interaction with the underlying surface. Scanning parameters: (a,b) $V_s = 0.15$ V, $I_t = 10$ pA. (c) $V_s = 0.15$ V, $I_t = 30$ pA. Scale bars: (a-c) 2nm.

8.5 Addition STS data of Tetraazanonacene

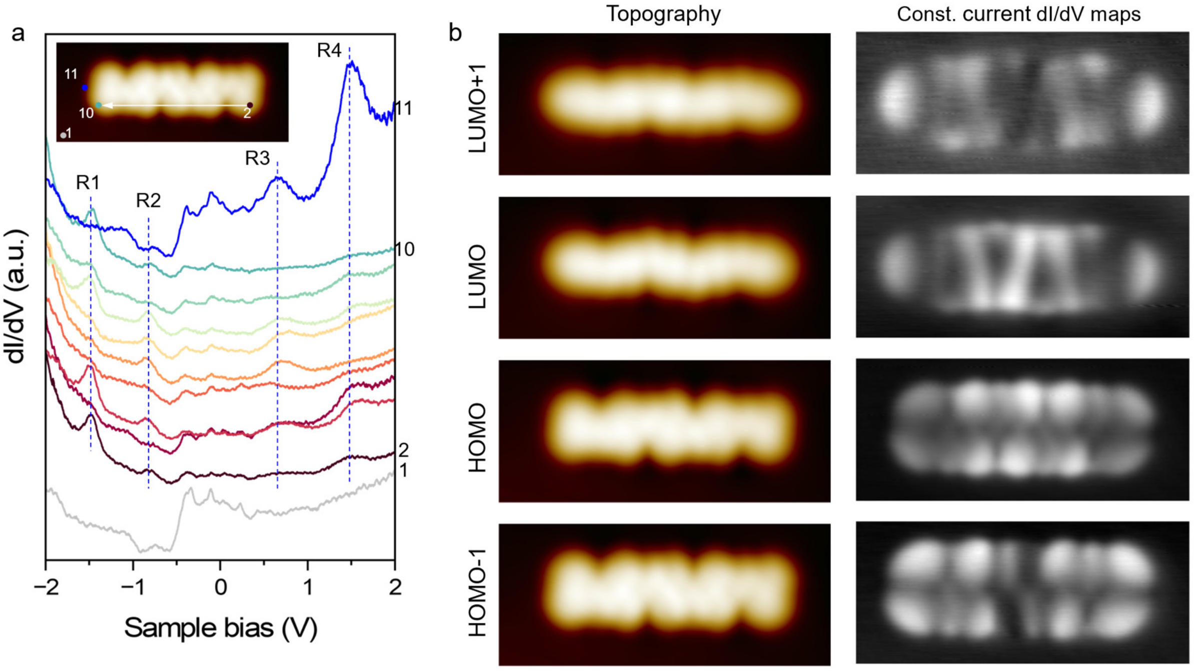

Figure S36. STS measurement of tetraazanonacene. (a) A series of dI/dV curves acquired at spots indicated in the inset. (b) constant current STM topographic images and constant current dI/dV maps of tetraazanonacene acquired at energies corresponding to peaks in (a). Scale bars: 1.5×3.5 nm for all images.

8.6 Calculated Relative Energies, Radical Character and HOMO-LUMO Gaps of Nonacene and Tetraazanonacene

Table S1. calculated properties of nonacene and tetraazanonacene for open-shell solutions

	tetraazanonacene	nonacene
$E_{CS\text{-}OS}$	0.27	0.18
y_0	0.863	0.754
y_1	0.317	0.295
$<S^2>$	1.23	1.11
$\Delta E_{H\text{-}L}{}^{OS}$	1.75	1.74
$\Delta E_{H\text{-}L}{}^{CS}$	1.04	1.17

Energies in eV

8.7 DFT calculated Spin Density of Tetraazanonacene

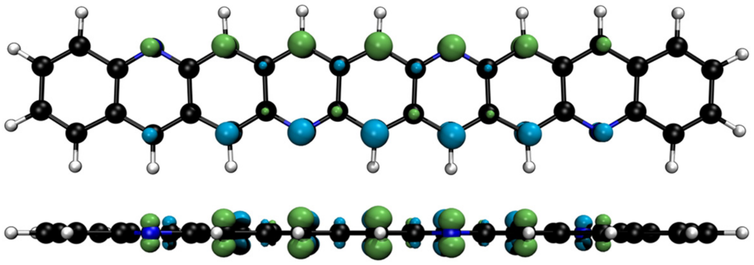

Figure S37. Electron spin density of tetraazanonacene.

8.8 CASSCF (14e,14o) Calculated $\Delta E_{S\text{-}T}$ and Natural Orbital Occupation Numbers of Tetraazanonacene and Nonacene

Table S2. CASSCF (14e,14o) calculated $\Delta E_{S\text{-}T}$ and natural orbital occupation numbers

	tetraazanonacene	nonacene
$\Delta E_{S\text{-}T}$	750 meV	401 meV
LUNO+2	0.109	0.338
LUNO+1	0.158	0.343
LUNO	0.253	0.553
HONO	1.755	1.448
HONO-1	1.839	1.603
HONO-2	1.886	1.716

8.9 DMRG-CASSCF(38e, 38o) Calculated Natural Orbital Occupation Numbers

Table S3. DMRG-CASSCF (38e,38o) calculated natural orbital occupation numbers

	tetraazanonacene	nonacene
LUNO+2	0.088	0.087
LUNO+1	0.127	0.132
LUNO	0.292	0.342
HONO	1.716	1.664
HONO-1	1.878	1.872
HONO-2	1.916	1.915

8.10 DMRG-CASSCF (38e, 38o) Calculated Unpaired Electron Density

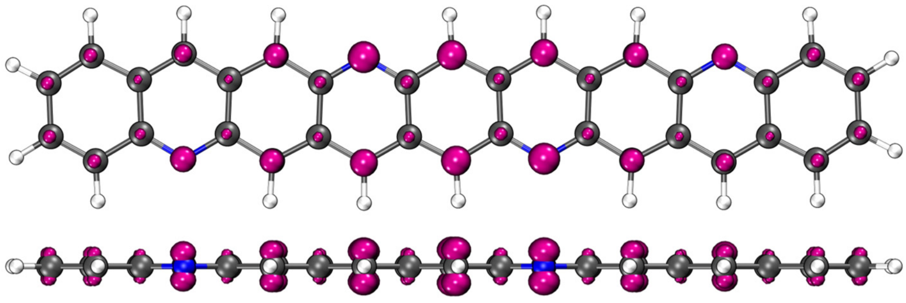

Figure S38. Unpaired electron density derived from DMRG-CASSCF calculations. Isosurfaces: 0.006 e/Å^3

8.11 Natural Orbitals of HONO-1, HONO, LUNO and LUNO+1 of Tetraazanonacene and Nonacene from DMRG-CASSCF(38e, 38o) Calculations.

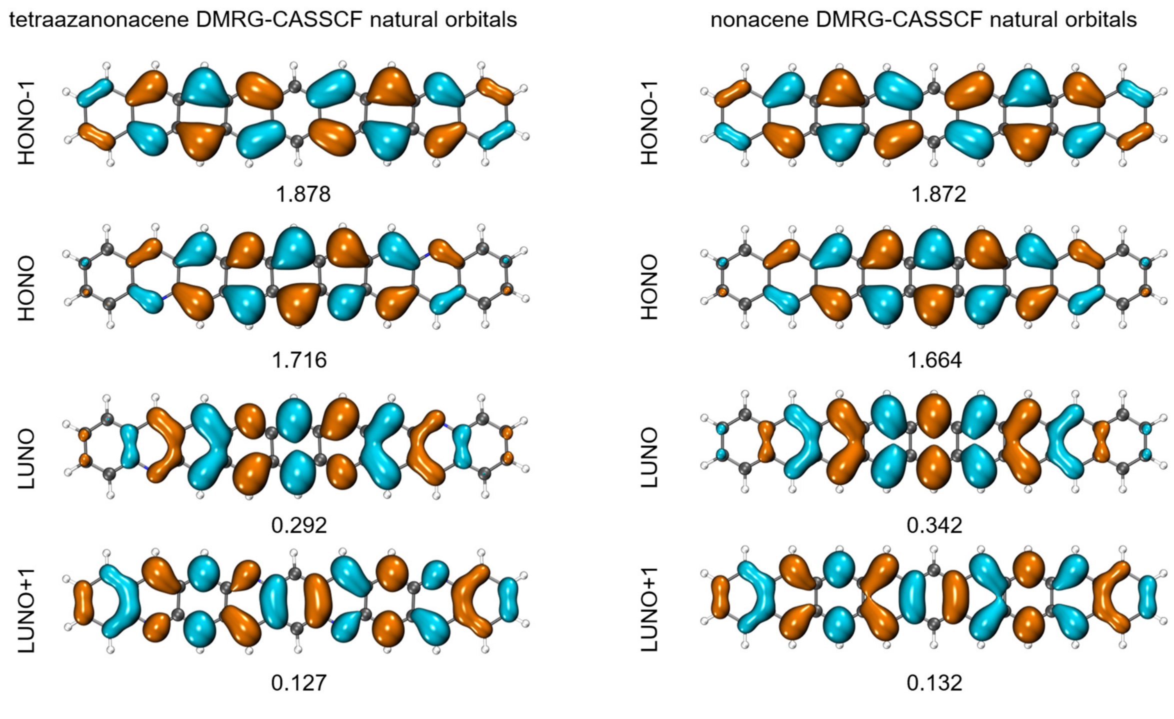

Figure S39. DMRG-CASSCF calculated natural orbitals of HONO-1, HONO, LUNO and LUNO+1, and their corresponding occupation numbers. Isosurface: 0.02 a.u.

8.12 Formation of Au-Tetraazanonacene Complex

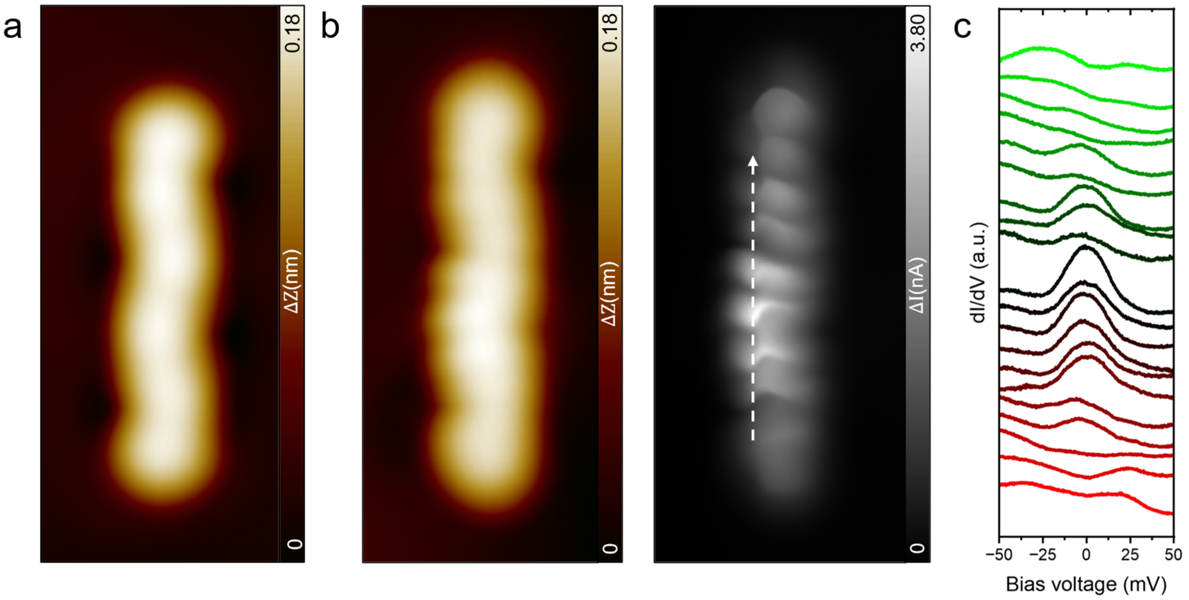

Figure S40. a. The same STM image as shown in Figure 2c in the main text, here show again for comparison. b. STM and BR-STM image of tetraazanonacene that bonded to the surface. c. The corresponding dI/dV curves near Fermi energy (2 mV), acquired along the dashed arrow in b). Scale bars: (a)1.5×3.5; (b,c) 1.5×3.2 nm.

8.13 Magnetically Induced Current Magnitude Density Plot

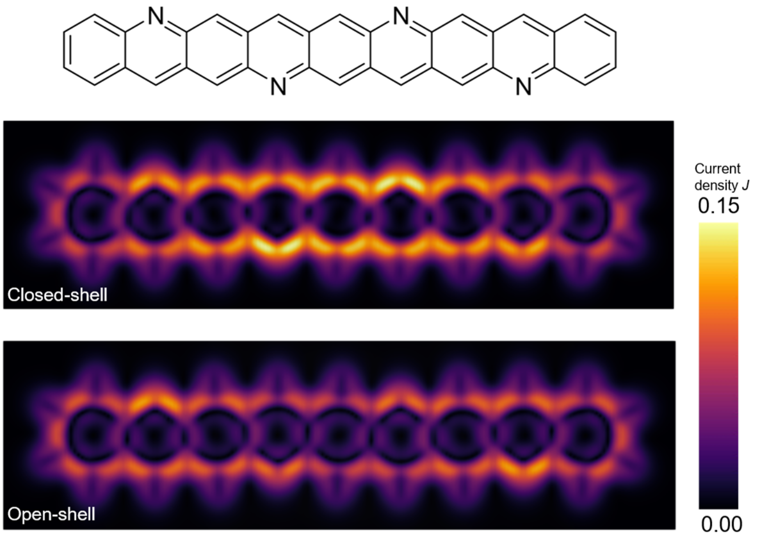

Figure S41. Magnetically induced current plot for a plane 1 Bohr above the molecule.

8.14 Magnetically Induced Current Magnitude Density Plot for Open-Shell Tetraazanonacene

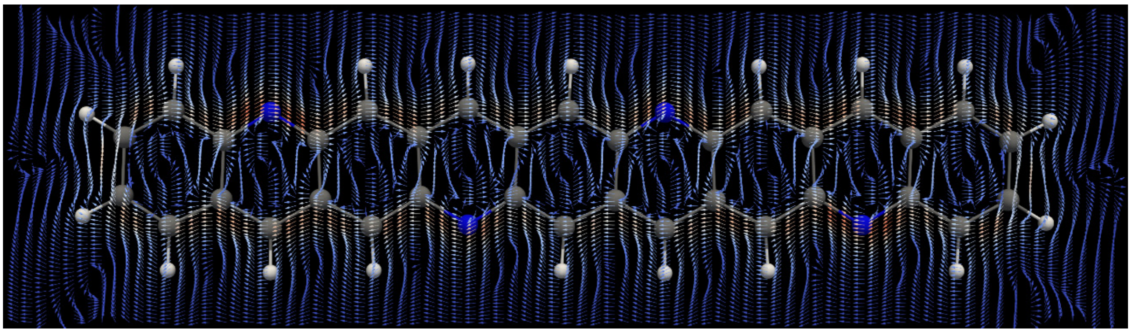

Figure S42. Magnetically induced current plot for a plane 1 Bohr above the molecule, the magnetically induced current is presented by arrows.

8.15 Calculated NICS$_{zz}$(1) Values for Difference Tetraazanonacene and its Analogues.

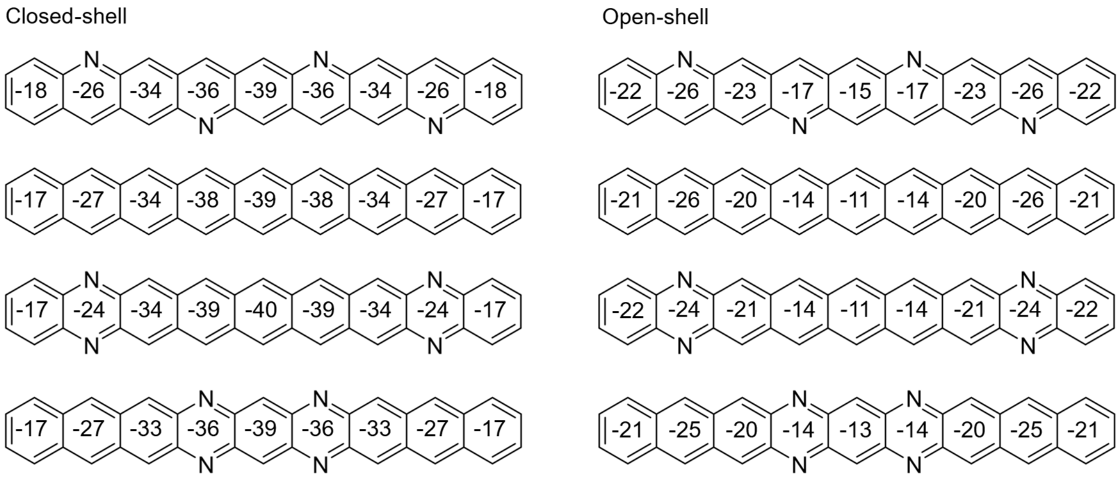

Figure S43. NICS$_{zz}$(1) values of three tetraazanonacene isomers and nonacene for the closed-shell (left) and open-shell (right) solution.

8.16 Calculated HOMO-LUMO Gaps and Frontier Orbitals for Tetraazanonacene and its Analogues for the Closed-Shell Solution

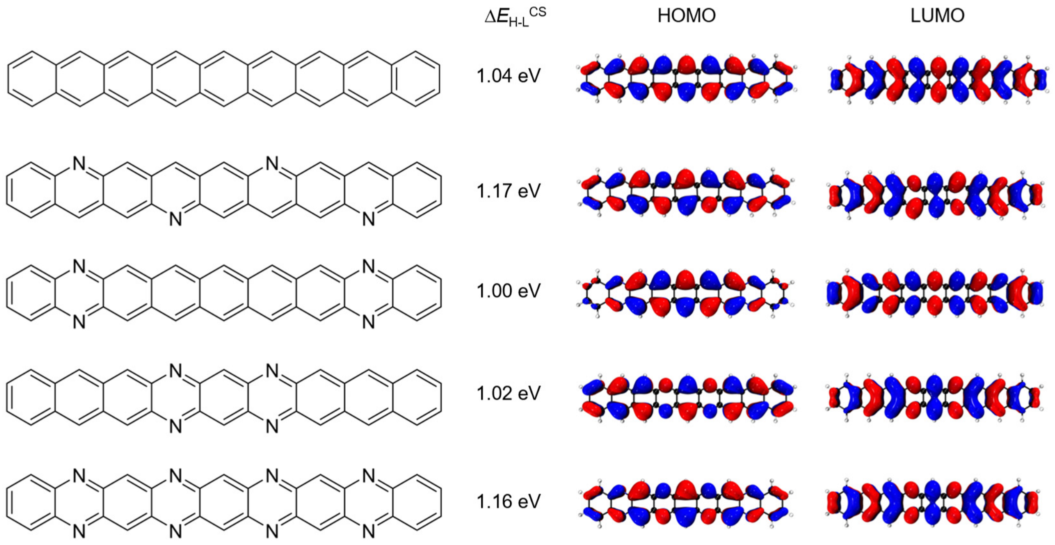

Figure S44. Calculated HOMO-LUMO gaps and frontier orbitals of nonacene, three tetraazanonacene isomers and octaazanonacene.

8.17 Comparison of the Aromaticity of Two Tetraazanonacene Isomers.

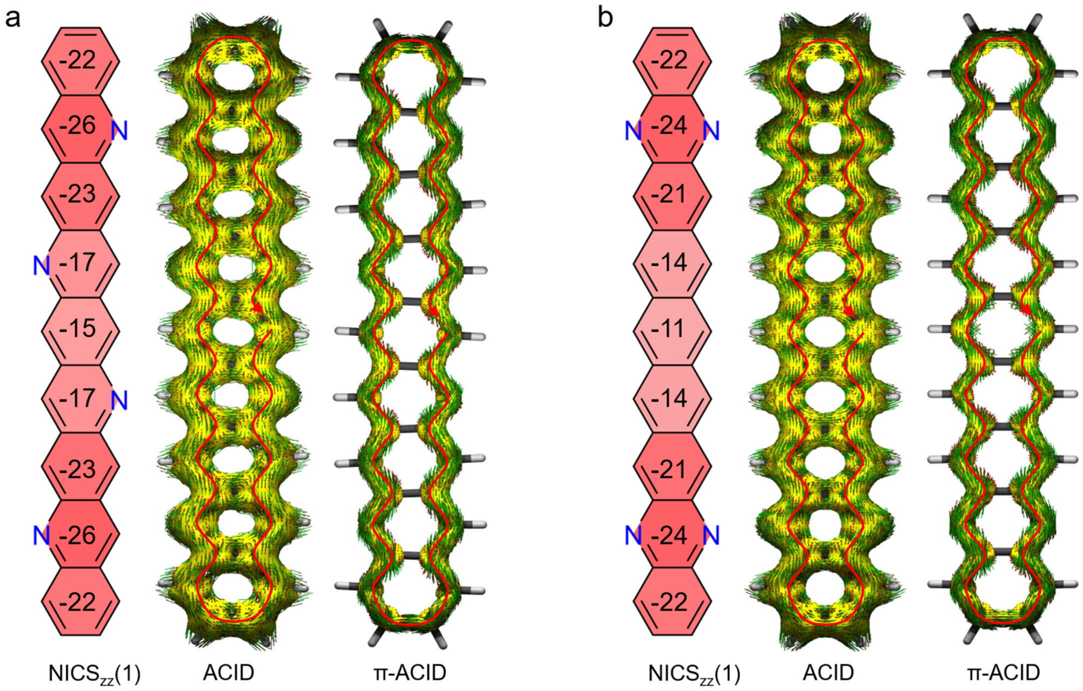

Figure S45. Calculated NICS$_{zz}$(1), ACID and π-ACID for (left) experimentally generated tetraazanonacene in open-shell state, same as shown in Figure 4, and (right) for its isomer.

8.18 Comparison of the Aromaticity of Tetraazanonacene and Tetraazaundecacene Reported in Ref. 23.

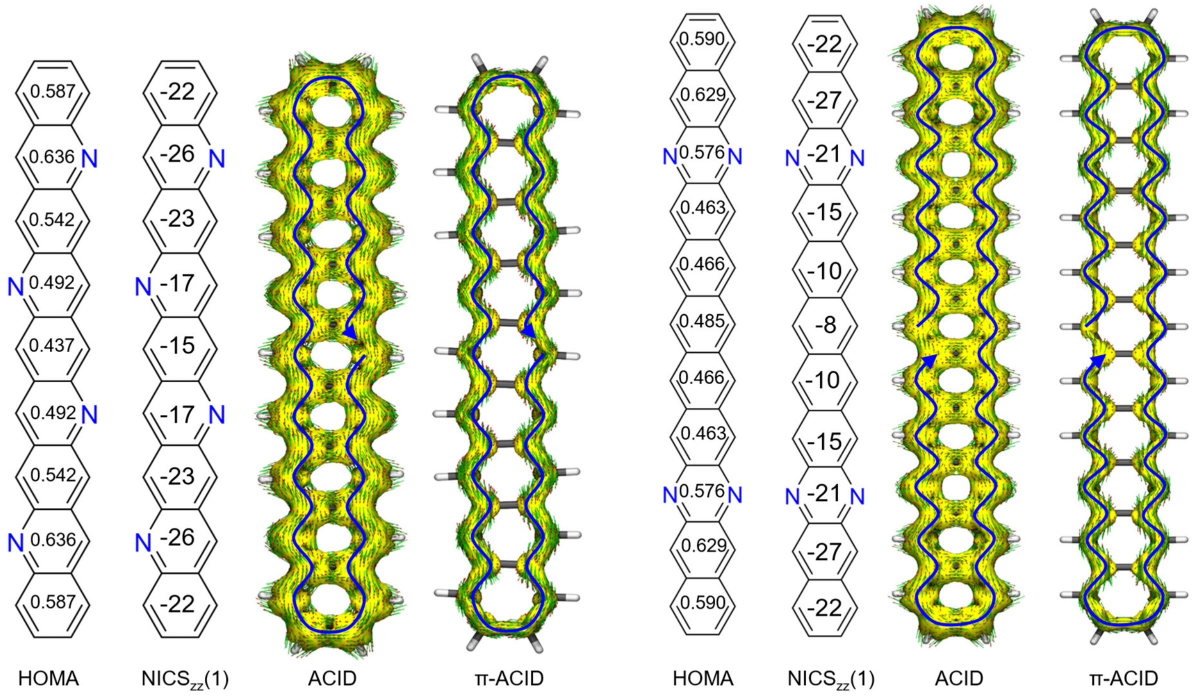

Figure S46. Calculated HOMA, $NICS_{zz}(1)$, ACID and π-ACID for (left) experimentally generated tetraazanonacene in open-shell state, same as shown in Figure 4, and (right) for tetraazaundecacene.
